# Proceedings of the 5th UK Implementation Science Research Conference

**DOI:** 10.1186/s13012-023-01270-7

**Published:** 2023-06-13

## Institute of Psychiatry, Psychology and Neuroscience, Kings College London

### P1 GERONTE Project: Development of a Framework to support implementation of Complex InterventiOns using Technology (CIo-uT): An Action Research study as part of multisite Randomised Controlled Trial

#### Bridget O’ Sullivan^1^, Anthony Staines^1^, Paul Davis^1^, Regina Connolly^1^, Trudy Corrigan^1^, Ciara White^1^, Shane O’ Hanlon^1,2^

##### ^1^Dublin City University, Ireland; ^2^St. Vincents University Hospital, Dublin, Ireland

###### **Correspondence:** Bridget O’ Sullivan (bridget.osullivan@dcu.ie)

*Implementation Science 2023*, **18(Suppl 1):**P1

**Background**

GERONTE is an EU funded project designed to improve the quality of life for older cancer patients with comorbidity by designing, implementing, and testing a novel technology-supported care pathway. Achieving efficiency and personalised care requires complex change to healthcare systems. Information Technology can support needed coordination (data sharing, communication, safety checks) on a large and sustainable scale. Implementing change into existing systems has high failure rates, due to patient and organisational-related complexity, highlighting the need for tailored, agile implementation plans. Implementing Science has established core theories and frameworks, but limited evidence on frameworks for complex interventions using technology.

**Method**

The aim is to co-create a framework to support widespread sustained implementation of the GERONTE intervention by identifying the: 1) intervention's mechanism of action; and the 2) contexts and strategies that impact implementation. An Action Research approach, using analysis and synthesis of qualitative and quantitative data, collected from the literature, and interviews, observation, and surveys with stakeholders, to co-design, test and refine the framework.

**Results**

The framework is at the co-creation stage, with analysis across stakeholders and contexts, to identify key factors that impact GERONTE's design, adaption, and implementation. The CLO-uT framework will build on, and apply, existing Implementation Science knowledge to support the implementation of innovative solution in line with changing healthcare needs and technological developments.

**Conclusion**

CIO-uT will provide a practical user-friendly framework to support the implementation of complex technology-supported interventions

GerOnTe: Streamlined Geriatric and Oncological evaluation based on IC Technology for holistic patient-oriented healthcare management for older multimorbid patients.

**Trial Registration:** Non applicable

**Acknowledgments:**

GERONTE is funded by the European Union’s Horizon 2020 research and innovation programme under grant agreement No. 945218.

**Consent to publish**

Non applicable

### P2 Co-design of an implementation plan for a digital holistic assessment and decision support framework for people with dementia care in care homes

#### Juliet Gillam^1^, Catherine Evans ^1,2^, Nathan Davies^3^

##### ^1^King’s College London, Cicely Saunders Institute of Palliative care, Policy & Rehabilitation, UK; ^2^Brighton General Hospital, Sussex Community NHS Foundation Trust, Brighton, UK; ^3^Centre for Ageing Population Studies, Research Department of Primary Care and Population Health, University College London, London, UK

###### **Correspondence:** Juliet Gillam (juliet.h.gillam@kcl.ac.uk)

*Implementation Science 2023*, **18(Suppl 1):**P2

**Background**

Positive findings around the use of eHealth to support dementia care in care homes are unfortunately insufficient to ensure its adoption in routine practice. A key strategy to promote uptake of eHealth is to co-design the intervention and implementation plan with users and relevant stakeholders. The aim of this study was to develop a plan with people with dementia, family carers and health and social care professionals to implement an eHealth intervention in care homes.

**Method**

An iterative co-design method was applied through a series of workshops which focused on co-developing implementation strategies, in response to identified determinants of implementation. Participants included family carers of people with dementia and practitioners with direct experience of working in care homes. A deductive thematic analytic approach was taken, guided by the constructs of the Normalisation Process Theory (NPT). Where data did not align, an inductive approach was taken.

**Results**

Implementation strategies which promoted the constructs of the NPT were selected. To target ‘coherence’, strategies focused on developing materials to promote the value of the eHealth intervention. ‘Cognitive participation’ was targeted through strategies which aim to maximise engagement with the intervention, including identifying champions and engaging care home managers. To promote ‘collective action’, strategies centered around maximising compatibility between routine practice and the intervention, and providing sufficient training and built-in user prompts. Strategies around ongoing adjustment and evaluation of the plan targeted ‘Reflexive monitoring’.

**Conclusion**

Implementing eHealth into such a complex system is a multifaceted process involving multiple stakeholders. Collaborating with stakeholders provided unique insight and perspective which can only be gained through lived-experience, and allowed us to co-develop a credible implementation plan with real world relevance. The theoretically informed strategies target the constructs of the NPT; mechanisms previously demonstrated to shape implementation process and outcomes. The plan is now ready for feasibility testing in care homes.

**Trial Registration**

Non applicable

**Consent to publish**

Non applicable

### O3 Maximizing knowledge from systematic reviews of complex interventions

#### Kristin J Konnyu^1^, Jeremy M Grimshaw^2^, Noah M Ivers^3^, Thomas Trikalinos^1^

##### ^1^Center for Evidence Synthesis in Health, Department of Health Services, Policy, and Practice, Brown University School of Public Health Providence, Rhode Island, USA; ^2^Centre for Practice-Changing Research (CPCR), Department of Medicine, University of Ottawa, Canada; ^3^Institute for Health System Solutions and Virtual Care (WIHV), Women's College Hospital, University of Toronto, Canada

###### **Correspondence:** Kristin J Konnyu (kristin_konnyu@brown.edu)

*Implementation Science 2023*, **18(Suppl 1):**O3

**Background**

Well-conducted randomized controlled trials (RCTs) are the gold standard for estimating intervention effects, and systematic reviews (SRs) of trial evidence are cornerstone to informing evidence-based practice, policy, and research. However, understanding the effects of complex interventions using standard SR approaches is challenging given the diversity of intervention content, delivered in diverse ways, evaluated in diverse designs using diverse outcomes. We describe methodological adaptations to standard review processes to enhance the informativeness of complex interventions SRs.

**Method**

The adaptations described are drawn from experiences in conducting 3 large SRs over the past 10 years.

**Results**

Question formulation - we adopted a modest and multivariable approach to inference. We assume true causal inference is not viable nor appropriate, but principled learning about associations between factors of interest and outcomes may be feasible. Data collection - contacting authors for additional details about interventions is feasible to supplement trial reports and authors are twice as likely to respond to requests if contacted by telephone vs email (1). Constructing a posterior distribution of intracluster correlation coefficients (ICC) is feasible and offers a principled approach to imputing missing ICCs among cluster RCTs that fail to account for unit of analysis errors (2). Data extraction – we have operationalized standardized taxonomies to code intervention content (3) to ensure robust (i.e., clinically or theoretically meaningful) coding. Data synthesis – we have found multivariable meta-regression models offer a feasible and informative approach to estimating the association between factors of interest and outcomes (4). Data reporting – we have adopted transparent reporting of our methods of data collection, manipulation, imputation, and analysis to complement the interpretation of our findings. We suggest complex interventions reviews are optimally suited to a living review framework (5).

**Conclusion**

Methodological adaptations of standard approaches may help enhance the informativeness of complex intervention SRs.

**Trial Registration:** Non applicable

**Consent to publish**

Non applicable

**References**

1. Danko KJ, Dahabreh IJ, Ivers NM, Moher D, Grimshaw JM. Contacting authors by telephone increased response proportions compared with emailing: results of a randomized study. Journal of Clinical Epidemiology. 2019 Nov; 115:150-159. PMID: 31152865
2. Konnyu KJ, Taljaard M, Ivers NM, Moher D, Grimshaw JM. Imputing intracluster correlation coefficients from a posterior predictive distribution is a feasible method of dealing with unit of analysis errors in a meta-analysis of cluster RCTs. J Clin Epidemiol. 2021 Jun 22:S0895-4356(21)00189-X. doi: 10.1016/j.jclinepi.2021.06.011. Epub ahead of print. PMID: 34171503.
3. Oatis CA, Konnyu KJ, Franklin PD. Generating consistent longitudinal real-world data to support research: Lessons from physical therapists ACR Open Rheumatology (In press).
4. Konnyu KJ, Grimshaw JM, Trikalinos TA, Ivers NM, Moher D, Dahabreh IJ. Evidence synthesis for complex interventions using meta-regression models. American Journal of Epidemiology (Under revision)
5. Elliott JH, Synnot A, Turner T, Simmonds M, Akl EA, McDonald S, Salanti G, Meerpohl J, MacLehose H, Hilton J, Tovey D, Shemilt I, Thomas J; Living Systematic Review Network. Living systematic review: 1. Introduction-the why, what, when, and how. J Clin Epidemiol. 2017 Nov;91:23-30. doi: 10.1016/j.jclinepi.2017.08.010. Epub 2017 Sep 11. PMID: 28912002.

### O4 Using behaviour change theory to assess intervention effectiveness in audit and feedback trials: A method for classifying and analysing interventions

#### Vivi Antonopoulou^1^, Carly Meyer^1^, Jacob Crawshaw^2^, Fabiana Lorencatto^1^, Justin Presseau^3^, Kristin Konnyu ^5^, Jesmin Antony^4^, Michelle Simeoni^4^, Susan Michie^1^, Jeremy Grimshaw^3^ & Noah Ivers^4^

##### ^1^Centre for Behaviour Change, Department of Clinical, Educational and Health Psychology University College London, UK; ^2^Centre for Evidence-Based Implementation (CEBI), Department of Medicine, McMaster University, Canada; ^3^Centre for Implementation Research, Ottawa Hospital Research Institute, Canada; ^4^Institute for Health System Solutions and Virtual Care (WIHV), Women's College Hospital, University of Toronto, Canada; ^5^Center for Evidence Synthesis in Health, Department of Health Services, Policy, and Practice, Brown University School of Public Health Providence, Rhode Island, USA

###### **Correspondence:** Vivi Antonopoulou (v.antonopoulou@ucl.ac.uk)

*Implementation Science 2023*, **18(Suppl 1):**O4

**Background**

Audit and feedback (A&F) is a frequently used quality improvement strategy to improve the implementation of evidence-based practice in healthcare. There is consistent evidence that A&F interventions deliver modest, variable, but significant improvements in clinical outcomes [1]. We are in the process of conducting an updated Cochrane review comprising 293 randomized trials of A&F. As part of this update, we examined intervention content to better understand which components are associated with greater effect sizes. We have used the behaviour change technique (BCT) taxonomy to content analyse the trials and leverage existing behaviour change theories to highlight key constructs relevant to A&F. The aim of the present study was two-fold: (1) to map key constructs of selected behaviour change theories relevant to A&F to BCTs; and (2) to describe the extent to which randomised trials of A&F incorporate theory-informed BCTs.

**Method**

We selected five behaviour change theories relevant to A&F: Goal Setting theory, Control theory, Feedback Intervention theory, Health Action Process Approach and Social Cognitive theory. For each theory, theoretical constructs were identified and linked to BCTs. For cross-validation, two separate processes were applied: theory experts cross-checked the BCT mapping onto constructs and A&F experts judged these BCTs for their relevance to A&F practice. Theory-informed BCTs were compared with BCTs identified in the analysis of the A&F trials included in the forthcoming Review.

**Results**

Preliminary results yielded 58 BCTs linked to constructs in one or more theories. The most frequently identified BCTs in theories were: ‘goal setting (behaviour)’, goal setting (outcome)’, ‘action planning’, ‘review behaviour goal’, and ‘review outcome goal’. In contrast, the most frequently identified BCTs in the A&F trials included in the review revealed 'feedback’, ‘instruction’, and 'social comparison’ to be the most frequently used.

**Conclusion**

Methodological considerations as well as implications for A&F research and practice will be discussed.

**Trial Registration:** Non applicable

**Consent to publish**

Non applicable

**References**

1. Jamtvedt G, Young JM, Kristoffersen DT, Thomson O’Brien MA, Oxman AD. Audit and feedback: effects on professional practice and health care outcomes. The Cochrane Database of Systematic Reviews [Internet]. 2003;(3):CD000259. Available from: https://pubmed.ncbi.nlm.nih.gov/12917891/
2. Ivers N, Jamtvedt G, Flottorp S, Young JM, Odgaard-Jensen J, French SD, et al. Audit and feedback: effects on professional practice and healthcare outcomes. Cochrane Database of Systematic Reviews [Internet]. 2012 Jun 13;(6). Available from: https://www.cochrane.org/CD000259/EPOC_audit-and-feedback-effects-on-professional-practice-and-patient-outcomes
3. Michie S, Johnston M. (2013). Behavior Change Techniques. In: Gellman MD, Turner JR, editors. Encyclopedia of Behavioral Medicine. New York: Springer; 2013. p. 182-187.

### P5 Implementation and dissemination of home and community-based interventions for informal caregivers of people living with dementia: a systematic scoping review

#### Eden M Zhu^1^, Martina Buljac-Samardžić^1^, Kees Ahaus^1^, Nick Sevdalis^2^, Robbert Huijsman^1^

##### ^1^School of Health Policy and Management, Erasmus University Rotterdam, Rotterdam, South Holland, 3062 PA, The Netherlands; ^2^Centre for Implementation Science, King’s College London, London, United Kingdom

###### **Correspondence:** Eden M Zhu (Zhu@eshpm.eur.nl)

*Implementation Science 2023*, **18(Suppl 1):**P5

**Background**

Informal caregivers of people with dementia (PwD) living at home are often the primary source of care, and, in their role, they often experience loss of quality of life. Implementation science knowledge is needed to optimize the real-world outcomes of evidence-based interventions (EBIs) for informal caregivers. This scoping review is the first to systematically synthesize the literature that reports implementation strategies employed to deliver home- and community-based EBIs for informal caregivers of PwD, implementation outcomes, and the barriers and facilitators to implementation in the research context.

**Method**

Embase, MEDLINE, Web of Science and Cochrane Library were searched from inception to March 2021; included studies focused on “implementation science”, “home- and community-based interventions” and “informal caregivers of people with dementia”. Titles and abstracts were screened using ASReview (an AI-based tool) and data extraction was guided by the ERIC taxonomy [1], the Implementation Outcome Framework [2], and the Consolidated Framework for Implementation Science Research [3]; each framework was used to examine a unique element of implementation.

**Results**

67 studies were included in the review. Multi-component (26.9%) and eHealth (22.3%) interventions were the most commonly found in included studies, and 31.34% of included studies were guided by an implementation science framework. Train and educate stakeholders and provide interactive assistance clusters had the most commonly employed implementation strategies, and acceptability (65.67%), appropriateness (70.14%) and penetration (58.21%) were the most frequently reported implementation outcomes. Design quality and packaging (intervention component suitability) and cosmopolitanism (partnerships) constructs, and patient’s needs and resources and available resources (infrastructure) constructs, contained the most frequently reported barriers and facilitators to implementation, respectively.

**Conclusion**

Future dementia studies must prioritize implementation science for more contextually-valid findings and examine how implementation partners can strategically leverage existing resources and regional networks to streamline local implementation. Mapping the evidence ecosystem will facilitate structured implementation planning.

**Trial Registration:** Non applicable

**Consent to publish**

Non applicable

**References**

1. Powell B, Waltz T, Chinman M, Damschroder L, Smith J, Matthieu M et al. A refined compilation of implementation strategies: results from the Expert Recommendations for Implementing Change (ERIC) project. Implementation Science. 2015;10(1).
2. Proctor E, Silmere H, Raghavan R, Hovmand P, Aarons G, Bunger A et al. Outcomes for Implementation Research: Conceptual Distinctions, Measurement Challenges, and Research Agenda. Administration and Policy in Mental Health and Mental Health Services Research. 2010;38(2):65-76.
3. Damschroder L, Aron D, Keith R, Kirsh S, Alexander J, Lowery J. Fostering implementation of health services research findings into practice: a consolidated framework for advancing implementation science. Implementation Science. 2009;4(1).

### P6 A co-creation approach to implementing eHealth applications in care organizations: lessons learned from multiple cases

#### Michel Oey^1^, Saskia Robben^1^, Margriet Pol^1^, Sanne Muiser^2^, Paulien Melis^2^, Somaya Ben Allouch^1^

##### ^1^Digital Life, Amsterdam University of Applied Sciences, Amsterdam, The Netherlands; ^2^Waag Society, Amsterdam, The Netherlands

###### **Correspondence:** Saskia Robben (s.m.b.robben@hva.nl)

*Implementation Science 2023*, **18(Suppl 1):**P6

**Background**

The uptake of eHealth technology in care organizations is low, considering the large supply of available eHealth applications. This study follows the implementation attempts of 18 European eHealth products (of several SME) in two health-care organizations to study the challenges during implementation, particularly in relation to acceptance, integration and scaling.

Experiences of health-care professionals were the primary focus of this study. This study is based on existing practical implementation guidelines [1], the five-phase model [2] and a co-creation approach.

**Method**

Three different sources were used to study implementation: 1) Four focus group sessions with health-care professionals, eHealth product developers, and policy makers within health-care organizations. 2) Informal and formal meetings of product evaluations. 3) Regular project meetings in a co-creation setting between all stakeholders to facilitate interaction.

**Results**

The result is a roadmap that provides guidelines and specific tips and tricks to both caregivers and developers to aid them in the implementation process. The roadmap added a “design and development” phase to the existing implementation model to emphasize the importance of co-creation for the implementation process. In addition to the implementation roadmap, an eHealth product catalog (both in a paper and digital version) and a guide to support the realization of business plans for SME have been developed.

**Conclusion**

The main conclusion of this study is the importance of engaging all the important stakeholders and to understand each other’s goals and needs. In addition, the co-creation approach was highly valued by the different stakeholders. Future work comprises studying the positive effects of this approach on improved eHealth applications, increased acceptance, and a smoother implementation process in care organizations.

**Trial Registration:** Non applicable

**Consent to publish**

Non applicable

**References**

1. ZonMW. Maak zelf een implementatieplan [Internet]. ZonMw Digitale Publicaties. [cited 2022 Oct 21]. Available from: https://publicaties.zonmw.nl/maak-zelf-een-implementatieplan/
2. Wensing MJP, Grol RPTM. Implementatie: Effectieve verbetering van de patiëntenzorg: Bohn Stafleu van Loghum; 2017.

### P7 The implementation and evaluation of a child weight e-learning toolkit (HealthyWEY) for maternity, health visiting and children’s centre workforces

#### James Harrison^1^, Julie Abayomi^2^, Shaima Hassan^3,4^, Lawrence Foweather^1^, Clare Maxwell^5^, Deborah McCann^1^, Sarah Garbett^1^, Maria Nugent^6^, Daisy Bradbury^7^, Hannah Timpson^8^, Lorna Porcellato^8^, Marian Judd^9^, Anna Chisholm^10^, Nabil Isaac^11^, Beth Wolfenden^6^, Amy Greenhalgh^6^, Paula M Watson^1^

##### ^1^Physical Activity Exchange, Research Institute for Sport and Exercise Sciences, Liverpool John Moores University, Liverpool, UK; ^2^Department of Allied Health and Social Care, Edge Hill University, UK; ^3^Department of Population Health Sciences, University of Liverpool, UK; ^4^NIHR Applied Research Collaboration NWC, Liverpool, UK; ^5^School of Nursing and Allied Health, Liverpool John Moores University, UK; ^6^Blackburn with Darwen Council, Blackburn with Darwen, UK; ^7^Sandwell and West Birmingham NHS Trust, UK; ^8^Public Health Institute, Liverpool John Moores University, UK; ^9^HCRG Care Group Services Limited, Salisbury, UK; ^10^Department of Psychology, Institute of Population Health, University of Liverpool, UK; ^11^Cornerstone Practice and Health Care, Blackburn with Darwen, UK

###### **Correspondence:** James Harrison (J.E.Harrison@2022.ljmu.ac.uk)

*Implementation Science 2023*, **18(Suppl 1):**P7

**Background**

With childhood obesity reaching epidemic levels [1], barriers to addressing the weight related behaviours of pre-school children have highlighted the need for appropriate training focused on increasing the knowledge, skills and confidence of healthcare professionals [2, 3]. Healthy Weight in Early Years (HealthyWEY) is an innovative training resource that brings together child weight-related information into a single e-learning package. This project explored the implementation of the HealthyWEY toolkit with multi agency workforces at 7 pilot sites across England, with the aim of assessing the effectiveness and feasibility of the resource for upskilling practitioners to support healthy weight-related behaviours during infancy and early childhood.

**Method**

Drawing on guidance provided in the MRC Process Evaluation Guide [4], a mixed-methods approach was used to assess the toolkit’s impact on health professionals’ knowledge, barriers, attitudes and motivations for addressing pre-school child weight, with focus groups to explore the acceptability of the e-learning and the barriers/facilitators to implementation. An embedded parent pilot was also conducted to assess the impact of HealthyWEY on parental knowledge and confidence to support a healthy weight in their child/ren.

**Results**

After engaging with the HealthyWEY e-learning, there were significant reductions in participants’ perceptions of the barriers to addressing pre-school child weight, significant increases in their perceived autonomy, competence and relatedness, and a significant increase in autonomous motivation for prioritising child weight management. The study’s findings also supported the acceptability of the e-learning among the multi-agency workforces at each pilot site. The impact of HealthyWEY was found to extend beyond the participating workforces, with a sample of parents/carers reporting increases in their knowledge and confidence to support a healthy weight in their child/ren following a consultation with a HealthyWEY-trained practitioner.

**Conclusion**

The project’s findings provide preliminary evidence of the toolkit’s effectiveness for upskilling multi-agency professionals to support healthy weight-related behaviours during infancy and early childhood.

**Trial Registration:** Non applicable

**Consent to publish**

Non applicable

**References**

1. NHS Digital. National Child Measurement Programme, England 2020/21 School Year. Available from: https://digital.nhs.uk/data-and-information/publications/statistical/national-child measurement-programme/2020-21-school-year
2. Ray D, Sniehotta F, McColl E, Ells L. Barriers and facilitators to implementing practices for prevention of childhood obesity in primary care: A mixed methods systematic review. Obesity Reviews. 2022; 1-14.
3. Turner GL, Owen S, Watson PM. Addressing childhood obesity at school entry: Qualitative experiences of school health professionals. Journal of Child Health Care. 2016; 20(3): 304-313.
4. Moore G, Audrey S, Barker M, Bond L, Bonell C, Cooper C, Hardeman W, Moore L, O’Cathain A, Tinati T, Wight D. Process evaluation of complex interventions: Medical Research Council Guidance. BMJ. 2015; 19;350: 1-7.

### P8 Associations between clinical and implementation outcomes of two psychoeducational programmes for type 1 diabetes in an effectiveness-implementation hybrid type 2 clinical trial

#### Tayana Soukup^1†^, Samantha Cross^1†^, Kia-Chong Chua^1^, Louise Hull^1^, Andy Healey^1^, Dulmini Kariyawasam^2^, Augustin Brooks^3^, Simon Heller^4^, Stephanie Amiel^5^, Kimberley Goldsmith^1^, Nick Sevdalis^1^, Ioannis Bakolis^1^, People with Diabetes Group^1^

##### ^1^Centre for Implementation Science, Health Service and Population Research Department, King’s College London; ^2^ Diabetes Department, Guy’s and St Thomas’ NHS Foundation Trust; ^3^Diabetes Department, Royal Bournemouth and Christchurch Hospitals NHS Foundation Trust; ^4^ Diabetes Department, Sheffield Teaching Hospitals NHS Foundation Trust; ^5^ Diabetes Department, King’s College Hospital NHS Foundation Trust

###### **Correspondence:** Tayana Soukup (tayana.soukup@kcl.ac.uk)

*Implementation Science 2023*, **18(Suppl 1):**P8

^†^These two authors share first authorship

**Background**

An established blood glucose awareness training (BGAT) was compared to a novel Hypoglycaemia Awareness Restoration Programme despite optimised care (HARPdoc) in a randomised hybrid trial [1,2]. While HARPdoc was not shown to be superior in reducing severe hypoglycaemia (SH) over 12 months, it was effective in reducing cognitive barriers to avoiding SH [3]. We report a comparative analysis of the implementation of HARPdoc to BGAT, and explore whether self-reported implementation outcomes are associated with clinical outcomes.

**Method**

This was an effectiveness-implementation hybrid type 2 trial (NCT02940873) occurring 2016-2021 in the UK and USA. Both BGAT and HARPdoc arms were rated for acceptability, appropriateness, and feasibility by the programme participants (n=45), their relatives (n=6), and healthcare providers (HCPs; n=27), totalling 48 assessments for HARPdoc and 41 for BGAT. Previously developed scales (AIM, IAM and FIM, respectively; 4 items each with a 5-point Likert scale) [4] were used for these implementation assessments.

Negative binomial regression with adjustment for baseline SH rates was used to examine the relationship between implementation scores and SH rates regardless of treatment. Linear regression was used to examine associations between implementation measures and treatment, and how implementation scores relate to clinical secondary outcomes (e.g., anxiety, depression), adjusted for those measures at baseline.

**Results**

All study participants rated HARPdoc higher on acceptability, appropriateness, and feasibility. Total implementation scores were significantly higher for HARPdoc (*M*=3.67, SD=0.80) than BGAT (*M*=4.22, SD=0.79) for the programme participants (difference=0.86, 95%CI:0.37–1.34, *p*=0.001) and for all participants (difference=0.55, 95%CI:0.22-0.89, *p*=0.01). A statistically insignificant 35% decrease in SH events at 12 months with each point increase in implementation rating was estimated. Secondary outcomes were inversely associated with higher implementation ratings.

**Conclusion**

We found evidence that programme participants, their relatives, and HCPs find HARPdoc more implementable than BGAT. This warrants further investigation of the implementability of the two programmes within a larger sample.

**Trial Registration:** NCT02940873

**Consent to publish**

Non applicable

**References**

1. Soukup T, Hull L, Smith EL, et al. Effectiveness-implementation hybrid type 2 trial evaluating two psychoeducational programmes for severe hypoglycaemia in type 1 diabetes: implementation study protocol. *BMJ Open*. 2019;9(11):e030370. Published 2019 Nov 14. doi:10.1136/bmjopen-2019-030370
2. Amiel SA, Choudhary P, Jacob P, et al. Hypoglycaemia Awareness Restoration Programme for People with Type 1 Diabetes and Problematic Hypoglycaemia Persisting Despite Optimised Self-care (HARPdoc): protocol for a group randomised controlled trial of a novel intervention addressing cognitions. *BMJ Open*. 2019;9(6):e030356. Published 2019 Jun 16. doi:10.1136/bmjopen-2019-030356
3. Amiel SA, Potts L, Goldsmith K, et al. A parallel randomised controlled trial of the Hypoglycaemia Awareness Restoration Programme for adults with type 1 diabetes and problematic hypoglycaemia despite optimised self-care (HARPdoc). *Nat Commun*. 2022;13(1):2229. Published 2022 Apr 28. doi:10.1038/s41467-022-29488-x
4. Weiner BJ, Lewis CC, Stanick C, et al. Psychometric assessment of three newly developed implementation outcome measures. *Implement Sci*. 2017;12(1):108. Published 2017 Aug 29. doi:10.1186/s13012-017-0635-3

### O9 A standardised method for the economic evaluation of implementation programmes: evaluating national programmes to increase the uptake of magnesium sulphate in pre-term births

#### Carlos Sillero-Rejon^1,2^, Hugh McLeod^1,2^, Brent C. Opmeer^1^, William Hollingworth^1,2^, Karen Luyt^3,4^

##### ^1^National Institute for Health Research Applied Research Collaboration West (NIHR ARC West) at University Hospitals Bristol and Weston NHS Foundation Trust. Whitefriars Level 9, Lewins Mead, Bristol, BS12NT, UK; ^2^Health Economics Bristol, Population Health Sciences, University of Bristol. Bristol, BS8 1UD, UK; ^3^Translational Health Sciences, Bristol Medical School, University of Bristol. 5 Tyndall Avenue, Bristol, BS8 1UD, UK; ^4^St. Michael’s Hospital, University Hospitals Bristol and Weston NHS Foundation Trust. Southwell Street, Bristol, BS2 8EG, UK

###### **Correspondence:** Carlos Sillero-Rejon (carlos.sillerorejon@bristol.ac.uk)

*Implementation Science 2023*, **18(Suppl 1):**O9

**Background**

Methods for the economic evaluation of implementation initiatives to increase the uptake of cost-effective healthcare interventions are not standardised. Value of implementation and policy cost-effectiveness are two proposed approaches. This research aims to demonstrate that these are mathematically equivalent and propose a standardised approach. To illustrate this, we evaluated two implementation programmes to increase magnesium sulphate uptake in preterm labour to reduce the risk of cerebral palsy: i) the National PReCePT Programme (NPP) which provided support and funded clinical time in maternity units in England, and ii) the PReCePT enhanced support model (ESP), which was nested within NPP in a cluster RCT.

**Method**

After summarising value of implementation and policy cost-effectiveness approaches, we show that they are mathematically equivalent, and propose a standardised stepwise method. We apply this method to the NPP (versus pre-existing trends) and the ESP (versus the NPP) calculating incremental cost-effectiveness ratios, net monetary benefits, and probabilities of being cost-effective.

**Results**

Estimating the cost-effectiveness of implementation programmes depends on the change in the healthcare technology uptake, cost of the implementation, size of the eligible population, and the cost-effectiveness of the healthcare technology. With our standardised stepwise analysis approach, the NPP cost £6,044 to implement per maternity unit and generated a societal lifetime net monetary benefit of £30,247 per unit over 12 months, at a willingness-to-pay threshold of £20,000; the probability of being cost-effective was 98%. In contrast, the ESP cost £16,869 to implement per unit and generated a net monetary benefit of -£28,682 per maternity unit in comparison to the NPP; the probability of being cost-effective was 22%.

**Conclusion**

Our standardised stepwise method enables the economic evaluation of implementation initiatives and is useful for implementation research. In this case, the NPP was highly cost-effective, but the addition of enhanced support was unlikely to be cost-effective.

**Trial Registration:** Non applicable

**Consent to publish**

Non applicable

### O10

#### Withdrawn

### O11 Barriers and facilitators to achieving co-production in care home settings: findings from a scoping review

#### Fran Hallam^1,2^, Katie Robinson^1, 2^, Meri Westlake^1, 2^, Pip Logan^2, 3^, Stephen Timmons^4^

##### ^1^Research and Innovation, Nottingham University Hospitals NHS Trust, UK; ^2^Centre for Rehabilitation and Ageing Research, Injury, Recovery and Inflammation Sciences, School of Medicine, University of Nottingham, UK; ^3^Nottingham CityCare Partnership, UK; ^4^Centre for Health Innovation, Leadership and Learning, Nottingham University Business School, University of Nottingham

###### **Correspondence:** Fran Hallam (frances.hallam@nottingham.ac.uk)

*Implementation Science 2023*, **18(Suppl 1):**O11

**Background**

Co-production involves the public, practitioners and academics working together as equals throughout all research stages [1]. Co-production may help to develop pragmatic, context-specific approaches to implementation which are acceptable to those living and working in care homes [2]. This scoping review aimed to map co-production approaches used in care homes for older adults in previous research, and to identify barriers and facilitators to achieving co-production in this context.

**Method**

The review was conducted following the Joanna Briggs Institute methodology for scoping reviews [3]. Seven databases were searched for published studies using co-production approaches in a care home setting. Studies were independently screened against eligibility criteria by two reviewers and citation searching was completed. Barriers and facilitators to co-production were synthesised using a deductive thematic analysis approach guided by the NIHR INVOLVE principles of co-production [1].

**Results**

19 studies were included. The focus and application of co-production approaches varied across the studies.11 studies reported barriers and 13 reported facilitators affecting the co-production process. Barriers and facilitators to building relationships and achieving inclusive, equitable and reciprocal co-production were identified in alignment with the five NIHR INVOLVE principles (Table 1). Practical considerations were also identified as potential barriers and facilitators.

**Conclusion**

The review has identified key factors which may influence authentic co-production in care home settings. The barriers and facilitators identified will inform the design of further research which aims to co-produce an implementation model for falls management in care homes.

**Trial Registration:** Non applicable

**Consent to publish**

Non applicable

**References**

1. National Institute for Health Research. Guidance on co-producing a research project. Available from: https://www.learningforinvolvement.org.uk/?opportunity=nihr-guidance-on-co-producing-a-research-project [Accessed 16 May 2022]
2. Peryer G, Kelly S, Blake J, Burton JK, Irvine L, Cowan A, et al. Contextual factors influencing complex intervention research processes in care homes: a systematic review and framework synthesis. Age and Ageing. 2022;51(3):afac014.
3. Peters MDJ GC, McInerney P, Munn Z, Tricco AC, Khalil, H. Chapter 11: Scoping Reviews (2020 version). In: Aromataris E, Munn Z (eds). JBI Manual for Evidence Synthesis. Joanna Briggs Institute; 2020.

**Table 1 (abstract O11).** Barriers and facilitators to achieving co-production in care homes

NIHR INVOLVE principle	Barriers	Facilitators
**Sharing power**	• Burden of supporting resident involvement on care staff • Gatekeeping • Ethical procedures • Delineating roles in the research process	• Creating opportunities to challenge dominant views • Reflexivity of project leads and researchers
**Including all perspectives and skills**	• Not enough involvement of key stakeholders • Pressures on care home staff and healthcare professionals • Care home resident characteristics • Limited depth of discussion • Difficulties with stretching perspectives	• Care home staff’s willingness to participate • Stimulating experiences • Flexible approach
**Respecting and valuing knowledge**	• Lack of self-confidence • Balancing different forms of knowledge	• Involvement across design stages • Recognising and utilising different forms of knowledge
**Reciprocity**	• Potential harms of participation	• Providing learning opportunities • Providing support • Clarifying expectations
**Building and maintaining relationships**	• Relationships with management • Differences between stakeholders • Optimising links with wider stakeholders • Practical challenges	• Project leaders and knowledge brokers • Building and utilising existing collaborative partnerships • Connection through creative approaches • Regular meetings and dialogue • Establishing ways of working • Sustaining relationship through participatory approach
**Other: Practical considerations**	• Feasibility of scaling co-production	• Logistical arrangements

### O12 Application of Normalisation Process Theory in the national scaling of early intervention for eating disorders

#### Katie L. Richards^1,2^, Karina L. Allen^1,3^, & Ulrike Schmidt^1,3^

##### ^1^Department of Psychological Medicine, King’s College London, Institute of Psychiatry, Psychology and Neuroscience, London, UK; ^2^Centre for Implementation Science, Health Service and Population Research Department, King’s College London, UK; ^3^Eating Disorder Outpatient Service, South London and Maudsley NHS Foundation Trust, London, UK

###### **Correspondence:** Katie L. Richards (katie.1.richards@kcl.ac.uk)

*Implementation Science 2023*, **18(Suppl 1):**O12

**Background**

Theories provide evidence-based and flexible tools to evaluate implementation processes. The Normalisation Process Theory (NPT) is a widely used implementation theory with demonstrated utility in supporting process evaluations [1]. This study evaluated the role of NPT mechanisms in the national implementation, embedding, and integration of an early intervention service for eating disorders.

**Method**

A mixed method evaluation was conducted. Twenty-one clinicians completed semi-structured interviews, and 211 clinicians completed longitudinal NPT questionnaires (NoMAD) administered before and after training and at a 3-month follow-up. For the qualitative data, the NPT was applied to inductively derived themes/subthemes to further evaluate underlying implementation mechanisms. The questionnaire data were analysed using multi-level growth models.

**Results**

The inductive thematic analysis yielded six themes and 15 subthemes outlining barriers and facilitators to implementation at the wider system, service, implementation strategy, intervention, clinician, and patient levels. The early intervention service was largely normalising in teams with high levels of sense-making, engagement, collection action, and appraisal work taking place. These NPT mechanisms were more evident for some subthemes (e.g., compatibility/integration) than others (e.g., patient complexity/comorbidities). Insufficient capacity was the main factor inhibiting the normalisation in services. The quantitative data paralleled the qualitative findings. Specifically, NPT mechanisms were high at the outset, especially ‘buy-in’ and engagement. The training led to significant improvements in the NPT subscales, which continued to improve or remained approximately the same at the 3-month follow-up. The exception to this were the items related to sufficient training and resources, which initially improved post-training, but reduced at the 3-month follow-up.

**Conclusion**

The NPT characterised key mechanisms that were shaped by and interacted with features of the early intervention service, implementation strategy, and context to facilitate or hinder implementation. However, not all aspects of the implementation were directly captured by the theory (e.g., patient complexity/comorbidity).

**Trial Registration:** Non applicable

**Consent to publish**

Non applicable

**References**

1. May C, Cumming A, Girling M, Bracher M, Mair F, May C, et al. Normalization Process Theory in feasibility studies and process evaluations of complex healthcare interventions: A systematic review. Implement Sci. 2018;13:80.

### O13 Use of routine healthcare data in randomised implementation trials: a methodological systematic review

#### Charis X. Xie^1^, Lixin Sun^2^, Elizabeth Ingram^3^, Anna De Simoni^1^, Sandra Eldridge^1^, Hilary Pinnock^4^, Clare Relton^1^

##### ^1^Wolfson Institute of Population Health, Queen Mary University of London, London, England, UK; ^2^School of Health and Related Research, University of Sheffield, Sheffield, England, UK; ^3^Department of Applied Health Research, University College London, London, England, UK; ^4^Asthma UK Centre for Applied Research, Usher Institute, The University of Edinburgh, Edinburgh, Scotland, UK

###### **Correspondence:** Charis X. Xie (charis.xie@qmul.ac.uk)

*Implementation Science 2023*, **18(Suppl 1):**O13

**Background**

Routine healthcare data are increasingly used in randomised controlled trials evaluating health interventions in participant identification, outcome assessment and intervention delivery [1]. Some trials evaluate the effect of strategies designed to improve the uptake of evidence-based practice (implementation trials) [2]. However, little is known about how routine data have been used in implementation trials. This review aims to describe the methodological characteristics, reported rationales, barriers and facilitators of randomised implementation trials conducted using routine data.

**Method**

We searched MEDLINE (Ovid), Cochrane Methodology Registry and Cochrane Central Register of Controlled Trials from Jan 2000 to Dec 2021, and manually searched protocols from trial registers. We included implementation trials and hybrid effectiveness-implementation trials [3] conducted using routine data. We extracted quantitative and qualitative data and narratively synthesised the findings.

**Results**

We included 80 implementation trials. Most evaluated multicomponent implementation strategies, as opposed to single strategies. The most frequently implemented evidence-based interventions were clinical guidelines. Most trials assessed adoption as the implementation outcome. The majority of trials used data from electronic health records in the combination of participant identification, intervention delivery and outcome assessment. The main rationales for using routine data were offering results validation, increasing efficiency, assessing outcomes, reducing research burden, improving quality of care, identifying study samples, and assessing representativeness. The most common barriers and facilitators were data quality, data delivery, EHR systems, research governance and external factors.

**Conclusion**

Identifying the implementation trials was difficult due to poor trial reporting. Further work is required to enhance the adoption of and adherence to existing guidelines on designing and reporting implementation studies [4, 5]. Additional work is needed to harmonise the language used in describing implementation strategies and implementation outcomes. Use of routine data is promising in implementation trials, future research should address barriers such as data quality to improve the employment of routine data.

**Systematic Review Registration:** PROSPERO CRD42022292321

**Trial Registration:** Non applicable

**Acknowledgments**

This work is funded by the Wellcome Trust [224863/Z/21/Z] and supported by the National Institute for Health Research ARC North Thames. The views expressed in this publication are those of the author(s) and not necessarily those of the National Institute for Health Research or the Department of Health and Social Care.

**Consent to publish**

Non applicable

**References**

1. Kwakkenbos L, Imran M, McCall SJ, McCord KA, Frobert O, Hemkens LG, et al. CONSORT extension for the reporting of randomised controlled trials conducted using cohorts and routinely collected data (CONSORT-ROUTINE): checklist with explanation and elaboration. BMJ. 2021;373:n857.
2. Bauer MS, Kirchner J. Implementation science: What is it and why should I care? Psychiatry Res. 2020;283:112376.
3. Curran GM, Bauer M, Mittman B, Pyne JM, Stetler C. Effectiveness-implementation hybrid designs: combining elements of clinical effectiveness and implementation research to enhance public health impact. Med Care. 2012;50(3):217-26.
4. Wolfenden L, Foy R, Presseau J, Grimshaw JM, Ivers NM, Powell BJ, et al. Designing and undertaking randomised implementation trials: guide for researchers. BMJ. 2021;372:m3721.
5. Pinnock H, Barwick M, Carpenter CR, Eldridge S, Grandes G, Griffiths CJ, et al. Standards for Reporting Implementation Studies (StaRI) Statement. BMJ. 2017;356:i6795.

### O14 Evaluation of the scale up of remote monitoring in rheumatology outpatients across three NHS trusts in London, UK

#### Helen Sheldon^1^, Kathryn Watson^2^, Rachel Olive^2^, Elena Pallari^1^, Camille Aznar^1^, Nikita Arumalla^3^, Olga Boiko^2^, Melanie Martin^3^, Len Demetriou^2^, Emily Jane Smith^3^, Emma-Jayne Adams^4^, Mary Ann Palmer^4^, Nick Sevdalis^2^, Andrew Walker^1^, Toby Garrood^3^

##### ^1^Health Innovation Network, London, SE1 9BB, UK; ^2^Centre for Implementation Science, Institute of Psychiatry, Psychology and Neuroscience, King’s College London, London, SE5 8AF, UK; ^3^Guy's and St Thomas' NHS Foundation Trust, London, SE1 9RT, UK; ^4^Lived experience study team members

###### **Correspondence:** Helen Sheldon (Helen.Sheldon3@nhs.net)

*Implementation Science 2023*, **18(Suppl 1):**O14

**Background**

Modern treat-to-target approaches to rheumatoid arthritis (RA) involve frequently monitoring disease activity via patient reported outcome measures (PROMs). Remote monitoring (RM) of PROMs can support care through more timely intervention and fewer unnecessary appointments. This study aimed to evaluate the feasibility of scaled implementation of a RM system for people with RA at three NHS trusts in London, UK.

**Method**

This was a prospective mixed-methods evaluation with service user involvement throughout. We report on the patient survey and semi-structured interviews with staff and patients exploring perspectives on the RM system. Interview schedule design and analysis for clinician and patient were informed by the EPIS [1] and COM-B [2] frameworks, respectively.

**Results**

Sixteen staff were interviewed. The system was implemented in two stages: an initial pilot at one trust then roll out to two other trusts. The four EPIS phases (Exploration, Preparation, Implementation and Sustainment) were evident in the pilot trust, but exploration and preparation were less evident at the other trusts. Adoption beyond the pilot trust was low with staff concerned about integration into clinical practice and systems.

Twenty-two patients were interviewed and 163 responded to the survey. Patients were overwhelmingly positive about the RM system. It was easy to use and required no skills beyond those used in their daily life. Patients were motivated to adopt the RM system by an interlinked set of beliefs regarding its use. A key motivator was increased responsiveness and ease of contact with the clinical service.

**Conclusion**

There was a contrast between the views of patients and staff outside of the pilot trust about RM. The lower adoption and associated concerns of staff about RM beyond the pilot site may be due to insufficient involvement at the Exploration and Preparation phases. The EPIS provides a useful framework for understanding challenges and approaches to scaling effectively.

**Trial Registration:** Non applicable

**Consent to publish**

Non applicable

**References**

1. Moullin JC, Dickson KS, Stadnick NA, Rabin B, Aarons GA. Systematic review of the Exploration, Preparation, Implementation, Sustainment (EPIS) framework. Implementation Science. 2019 Jan 5;14(1):1.
2. Michie S, van Stralen MM, West R. The behaviour change wheel: A new method for characterising and designing behaviour change interventions. Implementation Science. 2011 Apr 23;6(1):42.

### P15 Implementing patient-centered information tool to increase awareness and utilization of weight-loss surgery among obese Black men in the US

#### Katia Noyes^1,2^, Ajay A. Myneni^2^, Heather Orom^3^, Ranjit Singh^4^, Aaron Hoffman^2^

##### ^1^Division of Health Services Policy and Practice, Department of Epidemiology and Environmental Health, School of Public Health and Health Professions, University at Buffalo, Buffalo, New York, USA; ^2^Department of Surgery, Jacobs School of Medicine and Biomedical Sciences, University at Buffalo, Buffalo, New York, USA; ^3^Department of Community Health and Health Behavior, School of Public Health and Health Professions, University at Buffalo, Buffalo, New York, USA; ^4^Department of Family Medicine, Jacobs School of Medicine and Biomedical Sciences, University at Buffalo, Buffalo, New York, USA

###### **Correspondence:** Katia Noyes (enoyes@buffalo.edu)

*Implementation Science 2023*, **18(Suppl 1):**P15

**Background**

One size fits all implementation approach often results in implementation failure in marginalized communities. Obesity is one of the leading causes of preventable death in developed countries [1]. Minority patients bear a disproportionate burden of obesity but are less likely to receive surgical obesity treatment compared to Whites [2]. Evidence indicates that primary care providers (PCPs) rarely able to engage minority male patients in discussion about weight management [3]. The aim of this study is to identify culturally acceptable implementation strategies to disseminate accurate information about surgical weight management in minority men, to help men recognize their weight problem and its consequences, activate them to seek solutions, educate them about the safety and benefits of MBS, to help them locate a high-quality bariatric provider and receive insurance authorization.

**Method**

The study is conducted in partnership with our community advisory committee (CAC) consisting of stakeholders involved in care, services and decision-making for minority populations. Based on the input from the CAC, we design an educational tool using multiple iterative process obtaining feedback from community stakeholders. We pilot the tool among Black men (n=30) for final feedback and modify the tool to ensure cultural competency, effectiveness and acceptability of the end product. CAC and men are also asked about perceived effectiveness of different implementation strategies (e.g., a cartoon played by Black TV and radio stations vs in Black barber shops).

**Results**

Our study identified lack of role models for successful surgical weight loss as the most important barriers to Black men’s unwillingness to consider MBS. Black men expressed a strong preference for autonomy when making important health decisions and favored autonomy-preserving approaches to decision making.

**Conclusion**

New timely and effective strategies are needed to disseminate accurate information about surgical obesity management using patient-centered approaches as well as settings and social connections that patients trust.

**Trial Registration:** Non applicable

**Consent to publish**

Non applicable

**References**

1. Lewis KH, Edwards-Hampton SA, Ard JD. Disparities in Treatment Uptake and Outcomes of Patients with Obesity in the USA. Curr Obes Rep. Jun 2016;5(2):282-90. doi:10.1007/s13679-016-0211-1.
2. Hoffman AB, Myneni AA, Orom H, Schwaitzberg SD, Noyes K. Disparity in access to bariatric surgery among African-American men. Surg Endosc. Jun 2020;34(6):2630-2637. doi:10.1007/s00464-019-07034-z
3. Tork S, Meister KM, Uebele AL, et al. Factors Influencing Primary Care Physicians' Referral for Bariatric Surgery. JSLS. Jul-Sep 2015;19(3)doi:10.4293/JSLS.2015.00046

### P16 Integrating mental and physical healthcare: Evaluating the implementation of two novel interventions, Physical Health Clinic and Consultant Connect in a UK mental health NHS Trust

#### Theo Boardman-Pretty^1^, George Gillett^1^, Ray M^c^Grath^1,7^, Julie Williams^2^, Karen Ang^1,7^, Isabel McMullen^1^, Prashanth Reddy^3^, Fiona Gaughran^4^, Ioannis Bakolis^5^, Jorge Arias de la Torre^5^, Andy Healey^6^, Natalia Stepan^7^, Zarnie Khadjesari^8^, Euan Sadler^9^, Nick Sevdalis^2^ on behalf of the IMPHS study group

##### ^1^South London and Maudsley NHS Foundation Trust, London, UK; ^2^Centre for Implementation Science, King’s College London, London, UK; ^3^King's College Hospital NHS Foundation Trust, Denmark Hill, London, UK; ^4^Psychosis Studies, King's College London, London, UK; ^5^Department of Biostatistics and Health Informatics, King’s College London, London, UK; ^6^Kings Health Economics, King's College London, London, UK; ^7^Mind and Body Programme, King’s Health Partners, Guy’s Hospital, London, UK; ^8^Behavioural and Implementation Science (BIS) research group, University of East Anglia, Norwich, UK; ^9^Department of Nursing, Midwifery and Health, University of Southampton, Southampton, UK

###### **Correspondence:** Theo Boardman-Pretty (Theo.Boardman-Pretty@slam.nhs.uk)

*Implementation Science 2023*, **18(Suppl 1):**P16

**Background**

People with severe mental illnesses have poorer physical health and a reduced life expectancy compared to the general population. Two novel interventions, Consultant Connect (CC) and a Physical Health Clinic (PHC), were introduced in June 2020 at South London and Maudsley NHS Foundation Trust (SLaM) to improve integration between mental and physical healthcare systems and patient outcomes.

CC is an App that enables direct telephone access to specialist Consultants in local, acute hospitals for brief advice and guidance. All clinicians working at SLaM have access. The PHC is available to 12 adult mental health wards across SLaM. Referrers can request advice for various physical health complaints. A Consultant Physician responds by e-mail, telephone, or in person.

We report an ongoing prospective evaluation of the implementation and service impacts of the two interventions.

**Method**

Implementation of both interventions is being assessed by uptake data, validated measures of acceptability, appropriateness and feasibility and qualitative data collected via semi-structured interviews with users, using co-designed topic guides. A sample of users (n=10) will be interviewed per intervention. The ERIC implementation strategies framework will guide the assessment of implementation strategies for both interventions.

**Results**

From June 2020 to-date, CC has been used >1800 times; there have been >450 user downloads/registrations; >60 specialist services have been contacted. The PHC has received >80 referrals; from 35 referrers (32 medical / 3 nursing); from 12/12 inpatient wards included in the pilot. The above data are being mapped against the ERIC strategies to determine which strategies yielded higher uptake. Qualitative data collection is ongoing. We will update on our findings so far.

**Conclusion**

Integration of mental and physical health services is one potential approach to reduce the mortality gap in people with SMI. Our results can inform future service developments by providing insights into clinical and implementation effectiveness.

**Trial Registration:** Non applicable

**Consent to publish**

SLaM clinical governance and Information governance approvals

### P17 Development of the Implementation Science Research Project Appraisal Criteria (ImpResPAC) tool

#### Chloe Sweetnam^1^, Lucy Goulding^2^, Rachel Davis^2^, Zarnie Khadjesari^2,3^, Annette Boaz^4^, Andy Healey^2,5^, Nick Sevdalis^2^, Ioannis Bakolis^2,6^, Louise Hull^2^

##### ^1^Icahn School of Medicine at Mount Sinai, Neurology Department, New York, USA; ^2^Centre for Implementation Science, Health Service and Population Research Department, King’s College London, London, UK; ^3^School of Health Sciences, University of East Anglia, Norwich Research Park, Norwich, UK; ^4^London School of Hygiene & Tropical Medicine, London, UK; ^5^King’s Health Economics, Institute of Psychiatry, Psychology & Neuroscience, King's College London, London, UK; ^6^Department of Biostatistics and Health Informatics, Institute of Psychiatry, Psychology and Neuroscience, King's College London, London, UK

###### **Correspondence:** Chloe Sweetnam (chloe.sweetnam@mssm.edu)

*Implementation Science 2023*, **18(Suppl 1):**P17

**Background**

The need for quantitative criteria to appraise the quality of implementation research has recently been highlighted to improve methodological rigor [1]. The Implementation Science Research development (ImpRes) tool and supplementary guide provide methodological guidance and recommendations on how to design high-quality implementation research [2]. Here we report the development of the Implementation Science Research Project Appraisal Criteria (ImpResPAC) tool, a quantitative appraisal tool, developed based on the structure and content of ImpRes, to evaluate the conceptual and methodological quality of implementation research.

**Method**

This study employed a two-stage, prospective mixed-methods design. In stage 1, the 10 domains of the ImpRes tool, guidance and recommendations contained in the supplementary guide and within the literature, were mapped to ImpResPAC. In stage 2, an international multi-disciplinary expert group, recruited through purposive sampling, informed the refinement of ImpResPAC, including content, scoring system and user instructions. We also calculated descriptive characteristics for each domain.

**Results**

*Stage* 1:

We developed an initial version of ImpResPAC containing 55 items, indicating high-quality implementation research across 10 domains. ImpResPAC tool users assign a global score from 1-5 to each domain, indicating the quality of an implementation project.

*Stage 2:*

69 experts, from 8 countries, reviewed and provided feedback, including modifications and suggestions for improvement, on one or more ImpResPAC domains. Across 10 ImpResPAC domains, 50-75% of experts believe that the initial ImpResPAC domain items represented and reflected high-quality conceptual and methodological elements of implementation research. We are currently modifying ImpResPAC based on the extensive expert feedback we have received.

**Conclusion**

We have developed a quantitative appraisal tool, ImpResPAC, to allow implementation research stakeholders, primarily grant reviewers and educators, to undertake a comprehensive and transparent appraisal of the quality of implementation research. The next step of this research is to evaluate the psychometric properties of ImpResPAC.

**Trial Registration:** Non applicable

**Consent to publish**

Non applicable

**References**

1. Crable EL, Biancarelli D, Walkey AJ, Allen CG, Proctor EK, Drainoni ML. Standardizing an approach to the evaluation of implementation science proposals. Implementation Science. 2018 May 29;13(1).
2. Hull L, Goulding L, Khadjesari Z, Davis R, Healey A, Bakolis I, et al. Designing high-quality implementation research: Development, application, feasibility and preliminary evaluation of the implementation science research development (ImpRes) tool and guide. Implementation Science. 2019;14(1):1–20.

### P18 An Evaluation of Physical Healthcare within Adult Community Mental Health Teams at South London and Maudsley NHS Foundation Trust (SLaM)

#### Gracie Tredget^1^, Julie Williams^2^, Ray McGrath^1^, Karen Ang^1^, Fiona Gaughran^3^, Jorge Aria de la Torre^4^, Ioannis Bakolis^4^, Andy Healey^5^, Zarnie Khadjesari^6^, Euan Sadler^7^, Natalia Stepan^8^ and Nick Sevdalis^2^

##### ^1^South London and Maudsley NHS Foundation Trust, London, UK; ^2^Centre for Implementation Science, King’s College London, London, UK; ^3^Psychosis Studies, King's College London, London, UK; ^4^Department of Biostatistics and Health Informatics, King’s College London, London, UK; ^5^Kings Health Economics, King's College London, London, UK; ^6^Behavioural and Implementation Science (BIS) research group, University of East Anglia, Norwich, UK; ^7^Department of Nursing, Midwifery and Health, University of Southampton, Southampton, UK; 8 Mind and Body Programme, King’s Health Partners, Guy’s Hospital, London, UK

###### **Correspondence:** Gracie Tredget (gracie.tredget@slam.nhs.uk)

*Implementation Science 2023*, **18(Suppl 1):**P18

**Background**

People living with serious mental illnesses (SMI), such as Schizophrenia, are more likely to die prematurely (as much as 15-20 years earlier) from preventable physical health problems than the average population [1]. Despite this, little is known about how mental health staff perceive their role in providing physical healthcare, nor how these attitudes may impact upon patient care. We report a prospective pragmatic evaluation to explore perceptions, attitudes, and experiences of staff, patients, and carers, regarding physical healthcare within South London and Maudsley (SLaM) Adult Community Mental Health Teams (CMHTs). We aim to identify common barriers or facilitators that impact on clinical practice and patient experience and use insights to develop recommendations to improve future routine practice regarding physical healthcare.

**Method**

This is a prospective service evaluation in SLaM CMHTs using qualitative methodology. The evaluation involves semi-structured interviews (n=22), focus groups (n=42) and observations (n=10) with staff, patients, and carers. We aim to recruit 64 participants (40 clinical staff, 12 patients and 12 carers). The evaluation will focus on three areas: 1) attitudes, perceptions, and experiences, 2) physical health infrastructure (e.g., screening tools, equipment, patient data), and 3) knowledge, skills, and training. Framework analysis will be used to analyse and synthesise data collected across the data set. Findings will be reviewed via feedback workshops with participating staff to co-develop recommendations for SLaM.

**Results**

The data collection is ongoing. At the time of the conference, we will report on the evaluation methodology and share early findings.

**Conclusion**

This evaluation will provide insights into how staff in CMHTs deal with physical health and the main barriers and facilitators for staff, patients, and carers. We will use this to provide recommendations that can better support future routine physical health provision within community mental health services.

**Trial Registration:** Non applicable

**Consent to publish**

Non applicable

**References**

1. John A, McGregor J, Jones I, Lee SC, Walters JTR, Owen MJ, et al. Premature mortality among people with severe mental illness — New evidence from linked primary care data. Schizophrenia Research [Internet]. 2018 Sep;199:154–62. Available from: https://www.sciencedirect.com/science/article/pii/S0920996418301981

### P19 Tailoring strategies to support the implementation of Dose Adjustment For Normal Eating (DAFNE), a structured patient education programme for people with Type 1 diabetes

#### Fiona Riordan^1^, Claire Kerins^1^, Margaret Humphreys^2^, Sean Dinneen^3^, Luke Wolfenden^4^, Sheena M. McHugh^1^

##### ^1^School of Public Health, University College Cork, Cork, Ireland; ^2^Department of Medicine, Cork University Hospital, Wilton, Cork, Ireland; ^3^Centre for Diabetes Endocrinology and Metabolism, Galway University Hospital, Newcastle Road, Galway, Ireland; ^4^School of Medicine and Public Health, College of Health, Medicine, and Wellbeing, the University of Newcastle, Callaghan, NSW, Australia

###### **Correspondence:** Fiona Riordan (Fiona.riordan@ucc.ie)

*Implementation Science 2023*, **18(Suppl 1):**P19

**Background**

Evidence-based patient education programmes like DAFNE, which is prioritised for national implementation in Ireland, are recommended as part of diabetes management. However, little is known about current DAFNE implementation and how best to support delivery. Tailoring typically involves determinant identification, prioritisation, and selection of strategies, but how best to combine evidence, theory and stakeholder perspectives during prioritisation and selection is unclear [1,2]. To address this gap, we are 1) working with Irish DAFNE centres to tailor strategies, 2) evaluating the tailoring process, including how clinical stakeholders use evidence and guidance.

**Method**

To identify potential determinants, we (a) undertook a rapid review of structured diabetes education programmes and coded to CFIR (b) are analysing data from 91 Irish and UK DAFNE centres). DAFNE teams will complete a survey on their site characteristics (implementation culture, climate, readiness) before taking part in three group sessions to identify and prioritise determinants and select strategies. First, participants prioritise determinants and select strategies based on their own assumptions, needs and preferences. Then they will consider guidance (including feasibility of addressing a determinant, importance, ubiquity, chronicity, and criticality), determinant-strategy alignment of strategies, and evidence of strategy effectiveness. Participants’ experiences of the tailoring process will be evaluated via research logs, non-participant observation, surveys, and post-tailoring interviews.

**Results**

During 2019-2021 91 centres delivered 1257 courses (2 to 74 courses across centres) and 6749 people attended; 9.5% dropped out. Determinants identified included: lack of available resources (e.g., staff schedules), access to knowledge and information (e.g., staff preparation) and networking and communication (e.g., staff experience working with one another). For the next stage, we have invited 18 sites to participate in the tailoring process.

**Conclusion**

This study will advance our current understanding of tailoring, including clinical stakeholder decision-making during the process, and what is feasible and sustainable for them in practice.

**Trial Registration:** Non applicable

**Consent to publish**

Non applicable

**References**

1. Powell BJ, Beidas RS, Lewis CC, Aarons GA, McMillen JC, Proctor EK, et al. Methods to Improve the Selection and Tailoring of Implementation Strategies. The Journal of Behavioral Health Services & Research. 2015 Aug 21;44(2):177–94.
2. Wensing M, Grol R. Knowledge translation in health: how implementation science could contribute more. BMC Medicine. 2019 May 7;17(1).

### P20

#### Withdrawn

### P21 Expert consensus on multilevel implementation hypotheses to pro**m**ote uptake of youth care guidelines: A Delphi study

#### Evelien Dubbeldeman, Rianne van der Kleij, Evelyn Brakema, Matty Crone

##### ^1^Leiden University Medical Center, Department of Public Health and Primary Care, P.O. Box 9600, 2300 RC Leiden, The Netherlands

###### **Correspondence:** Evelien Dubbeldeman (e.m.dubbeldeman@lumc.nl)

*Implementation Science 2023*, **18(Suppl 1):**P21

**Background**

The implementation of evidence-based youth care guidelines remains a complex process. Several frameworks to aid the identification and specification of implementation determinants and effective strategies have been developed [1-4]. However, how specific determinants are influenced by specific strategies is not yet clear. There is a need for clarity on which active ingredients of strategies, called Behavior Change Techniques (BCTs) [5], elicit behavior change, and in turn, implementation outcomes. With this knowledge, we are able to formulated detailed, evidence-based implementation hypotheses. We aimed to identify 1) relevant determinants to the implementation of youth care guidelines and 2) feasible and effective implementation hypotheses to address these determinants.

**Method**

A four-round online Delphi study was conducted. In the first round, experts rated determinants on their relevance. In the second, implementation hypotheses were formulated by connecting BCTs and implementation strategies to determinants. In round three, experts reconsidered and finalized their hypotheses based on an anonymous overview of hypotheses formulated by all experts including their substantiations. Finally, experts were asked to rate the implementation hypotheses on potential effectiveness and feasibility.

**Results**

Fourteen experts completed the first, second, and third round and twelve the final round. Promotion of guideline use, Mandatory education, Presence of an implementation leader, Poor management support, Knowledge regarding use of the guideline, and Lack of communication skills were reported as most relevant. For each determinant, an overview is provided of the implementation hypotheses most often considered as effective and feasible.

**Conclusion**

Determinants related to knowledge, skills, and engagement of professionals and management were found to be relevant for the implementation of youth care guidelines.. This study provides a set of hypotheses that could facilitate organizations, policy makers, and professionals to guide the implementation process of youth care guidelines to, ultimately, improve implementation outcomes. Their effectiveness in practice remains to be assessed.

**Trial Registration:** Non applicable

**Consent to publish**

Non applicable

**References**

1. Cane J, O’Connor D, Michie S. Validation of the theoretical domains framework for use in behaviour change and implementation research. Implementation science. 2012;7(1):37.
2. Damschroder LJ, Aron DC, Keith RE, Kirsh SR, Alexander JA, Lowery JC. Fostering implementation of health services research findings into practice: a consolidated framework for advancing implementation science. Implementation science. 2009;4(1):1-15.
3. Nilsen P. Making sense of implementation theories, models, and frameworks. Implementation Science 30: Springer; 2020. p. 53-79.
4. Powell BJ, Waltz TJ, Chinman MJ, Damschroder LJ, Smith JL, Matthieu MM, et al. A refined compilation of implementation strategies: results from the Expert Recommendations for Implementing Change (ERIC) project. Implementation Science. 2015;10(1):1-14.
5. Michie S, Johnston M, Abraham C, Lawton R, Parker D, Walker A. Making psychological theory useful for implementing evidence based practice: a consensus approach. BMJ Quality & Safety. 2005;14(1):26-33.

### P22 Implementation strategies for an Australian school-based mental health prevention program: Realist evaluation

#### Rachel Baffsky^1,2^, Rebecca Ivers^1^, Patricia Cullen^1^, Michelle Torok^1^

##### ^1^University School of Population Health, UNSW Sydney, Samuels Building, F25, Samuel Terry Ave, Kensington NSW, Australia; ^2^Black Dog Institute, University of New South Wales, Hospital Road, Randwick NSW, Australia

###### **Correspondence:** Rachel Baffsky (r.baffsky@unsw.edu.au)

*Implementation Science 2023*, **18(Suppl 1):**P22

**Background**

The United Nations has issued a call to action for schools to deliver evidence-based prevention programs to address the growing burden of mental health, but implementation has failed in real-world settings. There is a need for implementation scientists to develop and trial strategies to address this translational problem.

**Method**

In this qualitative study, we used realist interviews and focus group discussions with educational staff (N=29) and performed a realist evaluation of a multicomponent implementation strategy called PAX Plus, designed to enhance the adoption of international evidence-based mental health prevention program, PAX Good Behaviour Game, in New South Wales primary schools.

**Results**

The PAX Plus strategies consistently reported to improve implementation outcomes were having a recognition system for positive reinforcement, leadership support through monthly meetings, training, and distributing support resources. Strategies that did not appear to work but could potentially be reformatted were monitoring progress using self-report methods, distributing e-newsletters with practical tips and having an online peer learning network.

**Conclusion**

Internationally, school-based practitioners can use findings from this study to develop/adapt their own strategies to improve the implementation outcomes of mental health prevention programs which will improve effectiveness outcomes. Improving the effectiveness of mental health prevention programs is a priority to address Sustainable Development Goal 3.4, to reduce premature death from non-communicable diseases by one third by 2030. This study also highlights to other implementation scientists how realist evaluations can be pragmatically used to improve knowledge translation of evidence-based programs in schools.

Learning Outcomes

We recommend school-based practitioners use recognition systems, training, leadership support and streamlined resources to increase the likelihood a mental health prevention program will be adopted and sustained in schools.

**Trial Registration:** Australian New Zealand Clinical Trials Registry, ACTRN12621001125819. Registered 23 August 2021 (version 1) – Retrospectively registered, https://anzctr.org.au/Trial/Registration/TrialReview.aspx?id=381346&isReview=true

**Consent to publish**

Written informed consent for publication was obtained.

### P23 Specifying and reporting implementation strategies used in the implementation of matrix support in mental health care in a medium-sized Brazilian city

#### Carlos Alberto dos Santos Treichel^1^; Ana Laura Salomé Lourencetti^2^; Maria Giovana Borges Saidel^2^; Rosana Teresa Onocko Campos^1^

##### ^1^Department of Collective Health, School of Medical Sciences, State University of Campinas, Campinas-SP, Brazil; ^2^School of Nursing, State University of Campinas, Campinas-SP, Brazil

###### **Correspondence:** Carlos Alberto dos Santos Treichel (treichelcarlos@gmail.com)

*Implementation Science 2023*, **18(Suppl 1):**P22

**Background**

Corresponding to a collaborative care proposal, for approximately 10 years matrix support has been consolidated as the Brazilian response to the need to integrate mental health services and primary care [1]. Although studies on its effectiveness are on the rise, to the best of our knowledge, studies focused on the strategies used for its implementation still missing. Thus, our objective was to specify and report the strategies used to implement matrix support in a medium-sized municipality.

**Method**

After the completion of an implementation process conducted between 2019 and 2021, participants of the Research Management Committee identified, through a consensus approach, the implementation strategies used to deliver the intervention. Strategies identification was supported by the taxonomy of implementation strategies proposed by the ERIC compilation [2], and their reporting followed the implementation strategy reporting guideline proposed by Proctor et al. (2013) [3].

**Results**

When reviewing the matrix support implementation process, twenty-four discrete implementation strategies were identified. Among the strategies used, those related to the development of relationships between stakeholders, training and education of stakeholders, and the use of evaluative and iterative strategies stood out. The strategies were mostly performed by research team members, managers and workers of local health services and members of partner universities. Strategies were used repeatedly at different times in the pre-implementation and implementation phases of the intervention and were mainly focused on characteristics of the inner context, characteristics of individuals and the implementation process. Among the implementation outcomes most affected by the strategies were acceptability, adoption, adequacy, and fidelity.

**Conclusion**

We believe that our work provides a source of knowledge that will allow other teams to envision implementation strategies that could be applied when undertaking efforts to implement matrix support in the context of mental health care in the future.

**Trial Registration:** Non applicable

**Acknowledgments**

This work was supported by the São Paulo Research Foundation (FAPESP) through grants n° 2018/10366-6 and 2020/14309-7.

**Consent to publish**

Non applicable

**References**

1. Treichel CA dos S, Campos RTO, Campos GW de S. Impasses e desafios para consolidação e efetividade do apoio matricial em saúde mental no Brasil. Interface - Comunicação, Saúde, Educação [Internet]. 2019 [cited 2021 Jul 29];23. Available from: https://www.scielo.br/j/icse/a/SMsPCj46yzmmjWJd83Vqx7J/?format=pdf&lang=pt
2. Powell BJ, Waltz TJ, Chinman MJ, Damschroder LJ, Smith JL, Matthieu MM, et al. A refined compilation of implementation strategies: results from the Expert Recommendations for Implementing Change (ERIC) project. Implementation Science. 2015 Feb 12;10(1).
3. Proctor EK, Powell BJ, McMillen JC. Implementation strategies: recommendations for specifying and reporting. Implementation Science [Internet]. 2013 Dec [cited 2019 Aug 15];8(1). Available from: https://www.ncbi.nlm.nih.gov/pmc/articles/PMC3882890/

### P24 Implementing telemedicine at scale in Denmark: Barriers and facilitators at the political-administrative level of the implementation process

#### Stina Bollerup^1,2^, Lotte Groth Jensen^1^, Camilla Palmhøj Nielsen^1,2^

##### ^1^DEFACTUM – Public Health & Health Services Research, Aarhus N, 8200, Denmark; ^2^Department of Public Health, Aarhus University, Aarhus C, 8000, Denmark

###### **Correspondence:** Stina Bollerup (stibol@rm.dk)

*Implementation Science 2023*, **18(Suppl 1):**P24

**Background**

Implementing technology and innovations on a large scale is a continuous challenge in healthcare systems [1]. This challenge is also seen in the case of telemedicine where implementation to practice and scale-up have proven difficult [2]. The political-administrative system plays a key role in the implementation process. Yet, this level of the implementation process remains understudied [3]. To address these gaps, this study will explore the political-administrative level of the national implementation process of TeleCOPD – a home-monitoring telehealth intervention targeting patients Chronic Obstructive Pulmonary Disease (COPD). Denmark is a pioneer country in regards to the implementation of telemedicine on a national scale [4]. This provides a unique chance to study large scale implementation of telemedicine and the role of contextual factors on the implementation process.

**Method**

An in-depth qualitative study of the implementation process at the political-administrative level will be undertaken. Data will be collected through semi-structured interviews with key stakeholders in the implementation process at the national, regional and municipality level. Furthermore, project descriptions and policy documents will be analysed to ascertain how the intervention is implemented across settings. Data will be analysed in accordance with thematic analysis.

**Results**

Reflections and preliminary results on how to investigate and theorize barriers and facilitators at a political-administrative level of the implementation process will be presented.

**Conclusion**

The results of this study will generate valuable knowledge about large scale implementation of telemedicine in addition to insights on the role of the political-administrative level in a implementation process.

**Trial Registration:** Non applicable

**Consent to publish**

Non applicable

**References**

1. Greenhalgh T, Papoutsi C. Spreading and scaling up innovation and improvement. BMJ. 2019;365:l2068.
2. Dinesen B, Nonnecke B, Lindeman D, Toft E, Kidholm K, Jethwani K, et al. Personalized Telehealth in the Future: A Global Research Agenda. J Med Internet Res. 2016;18:e53.
3. Leeman J, Baquero B, Bender M, Choy-Brown M, Ko LK, Nilsen P, et al. Advancing the use of organization theory in implementation science. Preventive Medicine. 2019;129:105832.
4. Nohr C, Villumsen S, Bernth Ahrenkiel S, Hulbaek L. Monitoring Telemedicine Implementation in Denmark. Stud Health Technol Inform. 2015;216:497-500.

### P25 Evaluating the implementation of Tommy’s Clinical Decision Tool, a device for reducing inequity in maternity care

#### Jenny Carter, Jane Sandall, on behalf of Tommy’s National Centre for Maternity Improvement

##### Department of Women and Children’s Health, Facility of Life Sciences and Population Health, King’s College London, London, UK

###### **Correspondence:** Jenny Carter (jenny.carter@kcl.ac.uk)

*Implementation Science 2023*, **18(Suppl 1):**P25

**Background**

Poor perinatal outcomes are more common in those living in areas of social deprivation and from ethnic minority groups. Causes of this disparity may be complex, but appear to include variation in care, as stillbirth and preterm birth rates vary between hospitals, even after adjustment for maternal characteristics. To address this variation in care, Tommy’s National Centre for Maternity Improvement developed the Tommy’s Clinical Decision Tool. This web-based tool assesses risk of preterm birth and placental dysfunction, which can lead to stillbirth, much more accurately than current methods, and recommends best evidenced-based care pathways in a format accessible to both women and healthcare professionals (HCPs). This study is evaluating implementation of the Tool in four early-adopter sites, to inform wider scale-up.

**Method**

Tommy’s Tool development, including determination of risk parameters and care pathways, involved maternity service users and HCPs in equal partnership. This study is evaluating: maternity service user and provider experience; barriers and facilitators to implementation; reach (whether particular groups are excluded and why), fidelity (degree to which the intervention is delivered as intended), and unintended consequences. Data is gathered through interviews, focus groups, questionnaires and through the Tool itself. The NASSS framework (Non-adoption or Abandonment of technology by individuals and difficulties achieving Scale-up, Spread and Sustainability) [1] is informing implementation and data analysis.

**Results**

Findings to date have informed ongoing developments of the Tool and implementation strategy, including those aimed at addressing digital and social exclusion (e.g. one-to-one support, language translation, animations). Other notable findings include: need for persistent, high-level local leadership, local champions, flexibility in training.

**Conclusion**

Tommy’s Tool has the potential to make providing “the right care at the right time” easier, personalising risk-assessment and care according to best evidence. Findings will inform implementation in scaling up in other settings.

**Trial Registration:** ISRCTN 13498237

**Consent to publish**

Non applicable

**References**

1. Greenhalgh T, Wherton J, Papoutsi C, Lynch J, Hughes G, Hinder S, Fahy N, Procter R, Shaw S. Beyond adoption: a new framework for theorizing and evaluating nonadoption, abandonment, and challenges to the scale-up, spread, and sustainability of health and care technologies. Journal of medical Internet research. 2017 Nov 1;19(11):e8775.

### P26 Implementing brief and low-intensity psychological interventions for children and young people: A rapid realist review

#### Anna Roach^1^, Sophie Cullinan^2^, Roz Shafran^1^, Isobel Heyman^1^, Sophie Bennett^1^

##### ^1^University College London Great Ormond Street Institute of Child Health, 30 Guilford Street, London, UK; ^2^ Institute of Education, University College London's Faculty of Education and Society, University College London, 20 Bedford Way, London, UK

###### **Correspondence:** Anna Roach (anna.roach.21@ucl.ac.uk)

*Implementation Science 2023*, **18(Suppl 1):**P26

**Background**

Despite research demonstrating that brief and low intensity psychological interventions are beneficial for children and young people with emotional, behavioural or mental health difficulties, there remains a significant implementation gap, leaving many children awaiting treatment. Innovative approaches are needed to develop, disseminate and implement appropriate psychological interventions [1].

**Method**

We conducted a rapid realist review to understand the barriers and facilitators to implementing brief or low-intensity psychological interventions in children and young people (PROSPERO protocol: CRD42022307367). We searched PsycInfo, EMBASE and Medline from inception to March 2022. Papers included in the review identified methods, factors and/or processes for the adoption, implementation or sustainability of brief and/or low intensity psychological interventions for children and young people (5-25 years) with emotional, behavioural or mental health difficulties. A systematic approach to data extraction using Normalisation Process Theory (NPT) [2] highlighted key barriers and facilitators.

**Results**

12 papers, including over 350 participants, met eligibility criteria. A variety of brief and/or low intensity psychological interventions were delivered across different settings by a range of individuals and common mechanisms were identified that promoted or impeded implementation. Personal, social, structural and organisational factors were all considered. Barriers included: 1) financial concerns, 2) capacity and time restraints and 3) staff turnover. Facilitators to implementation were 1) demonstrable economic benefit, 2) positive feedback from children and families and 3) specific individuals allocated to champion the intervention.

**Conclusion**

Our rapid realist review identified mechanisms and factors that need to be considered to optimise the implementation of brief and low-intensity interventions for children and young people with emotional, behavioural or mental health needs. Future research could consider creating a toolkit to help monitor and evaluate uptake into routine practice.

**Trial Registration:** Non applicable

**Consent to publish**

Non applicable

**References**

1. Wasil, AR, Park, SJ, Gillespie, S, Shingleton, R, Shinde, S, Natu, S, Weisz, JR, Hollon, SD, DeRubeis, RJ. Harnessing single-session interventions to improve adolescent mental health and well-being in India: development, adaptation, and pilot testing of online single-session interventions in Indian secondary schools. Asian journal of psychiatry. 2020; 50: -101980.
2. Murray, E, Treweek, S, Pope, C, MacFarlane, A, Ballini, L, Dowrick, C, May, C. Normalisation process theory: a framework for developing, evaluating and implementing complex interventions. BMC medicine. 2010; 8(1): -1-11.

### P27 Pragmatic and formative evaluation of the pilot implementation of UCLPartners’ Proactive Care Frameworks across multiple primary care sites in England

#### Alexandra Ziemann^1,2^, Zuhur Balayah^1^, Charitini Stavropoulou^1,3^, Katie Rose Sanfilippo^1^, Harry Scarbrough^1,4^, Matt Kearney^5^

##### ^1^Centre for Healthcare Innovation Research, City, University of London, London, UK; ^2^ Department of Social & Policy Sciences, University of Bath, Bath, UK; ^3^ School of Health and Psychological Sciences, City, University of London, London, UK; ^4^Bayes Business School, City, University of London, London, UK; ^5^ UCLPartners, London, UK

###### **Correspondence:** Alexandra Ziemann (alexandra.ziemann@city.ac.uk)

*Implementation Science 2023*, **18(Suppl 1):**P27

**Background**

The Academic Health Science Network (AHSN) UCLPartners developed the Proactive Care Frameworks (PCF) to support people with long term conditions during the pandemic and support the primary care system with post-pandemic recovery [1]. PCF consists of patient risk stratification/prioritisation, optimising workforce capacity and utilising digital resources to support self-management, remote support, and personalisation of care. In 2021, we evaluated the pilot implementation of PCF in six regions to derive insights informing ongoing implementation and spread efforts.

**Method**

The six-month pragmatic evaluation applied a mixed-method comparative case study approach. Guided by a Theory of Change, co-developed with implementation stakeholders, we assessed the impact of PCF implementation on care and work processes, workforce and patient/carer experience, health inequalities, and the implementation process. We analysed quantitative data from a survey among AHSNs and qualitative data from 41 implementation stakeholder interviews at AHSNs, local authorities, and general practices, and observations of nine Communities of Practice.

**Results**

Risk stratification supported clinicians to be more efficient and prioritise their work, freeing up time for higher skilled clinicians to see more complex patients. Staff reported an improved fit between patient needs and practice workforce, and increased patient knowledge, motivation and self-management skills. Critical learning included the need for realistic timeframes for implementation, dedicated implementation support, and sufficient engagement with both strategic leads and staff on the ground to allow for local adaptation and building ownership.

**Conclusion**

Rapid and pragmatic evaluation of early real-world implementation provided valuable formative insights to improve ongoing implementation. It also offered the opportunity to generate initial evidence about the potential impact of an innovation lacking an established traditional evidence base. Further rapid evaluation cycles should be conducted to gather direct patient/carer feedback, clinical and cost-effectiveness outcomes information, and identify core functions of PCF to improve local adaptation and spread.

**Trial Registration:** Non applicable

**Consent to publish**

Non applicable

**References**

1. UCLPartners. Proactive care frameworks [Internet]. Available from: https://uclpartners.com/proactive-care/. [Accessed 22 April 2022].

### O28 Evaluating Implementation Fidelity to a nurse-led model “INTERCARE”: A Mixed-Methods Study

#### Raphaëlle A. Guerbaai^1^, Sabina DeGeest^1,2^, Michael Simon^1^; Lori L. Popejoy^3^; Nathalie I. H. Wellens^4,5^, Kris Denhaerynck^1,2^, Franziska Zúñiga^1^

##### ^1^Department Public Health, Faculty of Medicine, Institute of Nursing Science, University of Basel, Basel, Switzerland; ^2^Public Health and Primary Care, Academic Centre for Nursing and Midwifery, KU Leuven, Leuven, Belgium; ^3^University of Missouri, Sinclair School of Nursing, Columbia, United States of America; ^4^Directorate General of Health, Department of Public Health and Social Affairs of the Canton of Vaud, 1014 Lausanne, Switzerland; ^5^La Source School of Nursing, HES-SO University of Applied Sciences and Arts Western Switzerland, 1004 Lausanne, Switzerland

###### **Correspondence:** Raphaëlle A. Guerbaai (RAPHAELLEASHLEY.GUERBAAI@UNIBAS.CH)

*Implementation Science 2023*, **18(Suppl 1):**O28

**Background**

Implementation fidelity assesses the degree to which an intervention is delivered as it should be. Little is known about how it acts as a moderator between an intervention and its intended outcome(s) and what elements affect the fidelity trajectory over time. We exemplify the meaning of implementation fidelity in INTERCARE, a nurse-led care model that was implemented in eleven Swiss nursing homes (NHs) with the aim of reducing unplanned hospital transfers. INTERCARE has six core elements that were introduced, among them advance care planning and tools to support inter- and intraprofessional communication.

**Method**

A mixed-methods design was used, guided by the Conceptual Framework for Implementation Fidelity. Fidelity to INTERCARE’s core components was measured with 44 self-developed items at 4 time points (baseline, 6, 12 months post intervention, 9 months post-intervention end); fidelity scores were calculated for each component and overall. Notes from NH meetings were used to identify moderators affecting the fidelity trajectory over time. Generalized linear mixed models were computed to analyze the quantitative data. Deductive thematic analysis was used for the qualitative analysis. The quantitative and qualitative findings were integrated using triangulation.

**Results**

A higher overall fidelity score showed a decreasing rate of unplanned hospital transfers post-intervention (OR: 0.65 (CI=0.43-0.99), p=0.047). Higher fidelity score to advance care planning was associated with lower unplanned transfers (OR= 0.24 (CI 0.13-0.44), p= < 0.001) and a lower fidelity score for communication tools (e.g., ISBAR) to higher rates in unplanned transfers (OR= 1.69 (CI 1.30-2.19), p= < 0.003).

**Conclusion**

High implementation fidelity to INTERCARE was necessary to achieve a reduction in unplanned transfers. In-house physicians with a collaborative approach and staff’s perceived need for nurses working in extended roles, were important factors supporting reaching high fidelity. Further research is needed to understand what supports the effective implementation of single elements.

**Trial Registration:** Non applicable

**Consent to publish**

Non applicable

### P29 Implementing guideline-based care in people with knee osteoarthritis: Development and evaluation of a patient education and self-management booklet in Tamil language

#### Devadhason Malarvizhi^1^, Dakshinamurthy Anandhu^1^, Jothi Suresh^1^, Devadhas Mercy Joy^1^, Thickvijayan S Veeragoudhaman^1^, Pakirisamy Maheshwari^2^, Cynthia S Srikesavan^3^

##### ^1^SRM College of Physiotherapy, Faculty of Medical and Health Sciences, SRM Institute of Science and Technology, Kattankulathur, Kancheepuram district, Tamil Nadu, India; ^2^Padmashree Institute of Physiotherapy, Bengaluru, Karnataka, India; ^3^Nuffield Department of Orthopaedics and Musculoskeletal Sciences, University of Oxford, United Kingdom

###### **Correspondence:** Devadhason Malarvizhi (malarvid@srmist.edu.in)

*Implementation Science 2023*, **18(Suppl 1):**P29

**Background**

Knee osteoarthritis (KOA) is a most common joint problem causing chronic joint pain, stiffness and loss of knee function [1]. KOA is managed by pharmacological and non-surgical treatments before surgery is considered. As per international guidelines [2-4], non-surgical treatments include patient education and self-management on exercises, pain coping strategies, weight reduction, and assistive devices and walking aids.

This study is part of an umbrella implementation project on a guideline-based and culturally-adapted KOA care for Tamil speaking people in Tamil Nadu state (population 77 million), South India. Our aim was to develop a patient education and self-management booklet in Tamil and evaluate its acceptability in routine clinical settings.

**Method**

A patient booklet was developed based on available research evidence and a needs assessment with patient representatives and physiotherapists. The booklet has simple text, exercise illustrations, photographs and a section on frequently asked questions by patients.

Preliminary evaluation was conducted in 50 adults with KOA, carers, and physiotherapists at the SRM medical college hospital and research centre in Kattankulathur, a sub-urban locality in Tamil Nadu. All participants provided signed consent and received a printed or digital booklet with instructions about using it. One week later, feedback was collected over the telephone using bespoke questionnaires.

**Results**

21 adults with KOA (4 males; 17 females; average age 59 years), 14 carers (7 males; 7 females; average age 50.3 years), and 15 physiotherapists (7 males; 8 females; average 18 years of work experience) participated.

Overall, participants found the booklet easily readable, useful and acceptable. They recommended some minor modifications to the wording for optimal clarity. A few further suggestions were to reorganise the exercises from easy to difficult levels and add specific exercise advice for the elderly.

**Conclusion**

Clinical benefits of the booklet will be evaluated in the next stage of this implementation project.

**Trial Registration:** Non applicable

**Consent to publish**

Non applicable

**References**

1. Eyles JP, Hunter DJ, Bennell KL, Dziedzic KS, Hinman RS, van der Esch M, Holden MA, Bowden JL, Quicke J, Skou ST, Risberg MA. Priorities for the effective implementation of osteoarthritis management programs: an OARSI international consensus exercise. Osteoarthritis and cartilage. 2019 Sep 1; 27(9):1270-9.
2. UK Nice Guidelines. Osteoarthritis: care and management in adults. [Internet]. 2022. Available from: https://www.nice.org.uk/guidance/cg177/resources/osteoarthritis-care-and-management-pdf-35109757272517
3. Bannuru RR, Osani MC, Vaysbrot EE, Arden NK, Bennell K, Bierma-Zeinstra SM, Kraus VB, Lohmander LS, Abbott JH, Bhandari M, Blanco FJ. OARSI guidelines for the non-surgical management of knee, hip, and polyarticular osteoarthritis. Osteoarthritis and cartilage. 2019 Nov 1; 27(11):1578-89.
4. Ministry of Health & Family Welfare, Government of India. Standard treatment guidelines: Management of Osteoarthritis Knee. 2017.

### O30 De-implement, Adapt, Reinvest and Evaluate; introducing the DARE Framework to deliver higher value healthcare

#### Jack J Bell^1^, Tracey Brighton^1^, Tamlyn Rautenberg^2,3^, Nina Meloncelli^2^

##### ^1^Allied Health, The Prince Charles Hospital, Chermside 4032, Australia; ^2^Allied Health, Metro North Health, Herston, 4006, Australia; ^3^Centre for Applied Health Economics (CAHE), Griffith University, Nathan, 4111, Australia

###### **Correspondence:** Jack J Bell (jack.bell@health.qld.gov.au)

*Implementation Science 2023*, **18(Suppl 1):**O30

**Background**

Many implementation theories, models and frameworks support implementation of health service innovations, and growing attention is directed towards de-implementation approaches. However, an integrated framework that supports pragmatic de-implementation, adaptation, reinvestment and evaluation remains lacking.

This initiative aimed to co-design a framework and toolkit to support de-implementation to reinvest approaches to improve health and care outcomes.

**Method**

The knowledge-to-action framework underpinned development of the DARE Framework and toolkit for feasibility testing in a convenience sample of allied health services in a single metropolitan hospital.

**Results**

An initial conceptual framework included synthesised concepts from underlying theories (n=3), process models (n=5), determinant (n=5) and evaluation frameworks (n=3) in August 2021. Iterative co-design with stakeholders (clinicians and managers) between August and October, 2021 applied data from twenty-four nominal group technique workshops, and 3 semi-structured focus groups. Findings were triangulated using informal group discussions, interviews and meetings to engage stakeholders in the iterative development, implementation, and refinement of the model and toolkit. Full consensus for facilitated rapid action cycle implementation and pragmatic feasibility testing of the draft model across allied health services for a 700+ bed hospital was achieved in November 2021 in response to unsustainable budgetary and service needs. At time of abstract preparation, RE-AIM evaluation demonstrates ongoing iterative adaptation of the model and toolkit, willingness to update and spread to medical and nursing professions, adoption, implementation and embedding of ranked de-implementation and reinvestment opportunities across all core allied health services in the test site. Limited effectiveness testing to date across process measures and quadruple aim healthcare outcomes appears strongly favourable; detailed findings will be presented at the conference as a qualitative case series.

**Conclusion**

Early data supports consideration of the DARE Framework as a useful approach to support rapid cycle, de-implement to reinvest approaches that deliver higher value health care.

**Trial Registration:** Non applicable

**Consent to publish**

Non applicable

### P31 Exploration of barriers and facilitators to the implementation of ventilator bundle: a descriptive qualitative study with health care professionals, Nepal

#### Dejina Thapa^1^, Ting Liu^1^, Chen Yang^1^, Subhash Prasad Acharya^2^ and Sek Ying Chair^1^

##### ^1^The Nethersole School of Nursing, Faculty of Medicine, The Chinese University of Hong Kong, Shatin, N.T., Hong Kong SAR, The People’s Republic of China; ^2^ Department of Anesthesiology, Tribhuvan University, Institute of Medicine, Kathmandu, Nepal

###### **Correspondence:** Dejina Thapa (dejinathapa@link.cuhk.edu.hk)

*Implementation Science 2023*, **18(Suppl 1):**P31

**Background**

Low- and middle-income countries, like Nepal, have greater rates of ventilator-associated pneumonia than high-income countries [1]. Effective implementation of ventilator bundle is crucial to reduce the occurrence of ventilator-associated pneumonia [2]. So far, no comprehensive assessment of barriers to sustained, successful implementation of hospitals interventions has been conducted in Nepalese healthcare settings. The main aim of the study is to identify the perceived barriers and facilitators of health care professionals to the implementation of the ventilator bundle. The result of the study will help to develop a tailored made intervention to maximize the adoption of the guidelines in Nepal.

**Method**

This qualitative study used the semi-structured virtual interview, enrolled twenty-one participants; nurses (n=18) and doctors (n=3) were selected by purposive sampling. The study setting was a general ICU and medical ICU at a tertiary academic hospital, Nepal. All the interview data were transcribed, coded, using thematic analysis, and analysed using the NVivo software.

**Results**

Provider-related factors, organisational, environmental, and patient factors were the major identified barriers that could affect the implementation of the ventilator bundle. The major barriers were a high rate of nursing turnover, imbalanced nurse-to-patient ratio, heavy workload, time spent on training new employees, lack of knowledge and skills, especially in novice nurses, and lack of motivation and reward. The key facilitators were timely educational training and workshops, ensuring the availability of strong leadership and champions, and providing adequate support at the organisational level.

**Conclusion**

The findings of this qualitative study revealed that organisational support is critical to the effective implementation of the guidelines. Building on these facilitators and addressing and measuring these barriers may aid in improving the acceptability and sustainability of the ventilator bundle especially among the nurses.

**Trial Registration:** Non applicable

**Consent to publish**

Non applicable

**References**

1. Bonell A, Azarrafiy R, Huong VTL, et al. A systematic review and meta-analysis of ventilator-associated pneumonia in adults in Asia: an analysis of national income level on incidence and etiology, Clinical Infectious Diseases 2019;68:511-8.
2. Klompas M, Branson R, Eichenwald EC, et al. Strategies to prevent ventilator-associated pneumonia in acute care hospitals: 2014 update, Infect Control Hosp Epidemiol 2014;35:915-36.

### O32 Using rapid qualitive inquiry for implementation support in a multinational study on infection prevention and control in neonatal intensive care

#### Emanuela Nyantakyi^1^, Marie-Therese Schultes^1^, Julia Bielicki^2,3^, Tuuli Metsvaht^4^, Lauren Clack^1,5^, & the NeoIPC consortium

##### ^1^Faculty of Medicine, Institute for Implementation Science in Health Care, University of Zurich, Zurich, 8006, Switzerland; ^2^Paediatric Infectious Diseases Research Group, St George's University of London, London, SW170RE, United Kingdom; ^3^ Paediatric Research Centre UKBB, University Children’s Hospital Basel, Basel, 4056, Switzerland; ^4^ Department of Paediatrics, Institute of Clinical Medicine, Tartu University Hospital, Tartu, 50406, Estonia; ^5^ Department of Infectious Diseases and Hospital Epidemiology, University Hospital Zurich, Zurich, 8091, Switzerland

###### **Correspondence:** Emanuela Nyantakyi (emanuela.nyantakyi@uzh.ch)

*Implementation Science 2023*, **18(Suppl 1):**O32

**Background**

The EU Horizon 2020 project NeoIPC aims to identify effective infection prevention and control interventions and corresponding implementation strategies for neonatal intensive care units (NICUs). In preparation of the trial, an implementation needs assessment survey with participating units in several European countries and South Africa was conducted. In the meantime, concerns among health professionals regarding the safety of the planned intervention and study design became apparent. A rapid qualitative approach was chosen to better understand these concerns and inform ongoing trial preparation.

**Method**

The survey was disseminated online to 22 participating NICUs and collected information regarding barriers and facilitators to the planned intervention based on scenarios with open response options. Two virtual focus groups (FGs) à 90 minutes were held. The FGs were centered around the relevance, efficacy, and safety of the planned intervention and potential concerns regarding the conduct of cluster randomized controlled trials (cRCTs) in NICUs. To quickly integrate the results into the project, data collection and analysis in both assessments were guided by a rapid qualitative approach using the CFIR framework based on [1].

**Results**

Thirteen NICUs responded to the survey. The FGs were attended by nine pediatricians and neonatologists from six European countries. In both assessments, the evidence base for the planned intervention and aspects of its compatibility with routine practice were deemed primary barriers. Stakeholder engagement strategies were named as potential facilitators to implementation. Including nurses to determine feasibility (i.e., practice fit) of interventions was suggested in the FGs. No concerns regarding the conduct of cRCTs were raised.

**Conclusion**

In our study, a pragmatic qualitative approach of rapid data assessment and analysis provided valuable information to implementation design and project development. However, the homogeneity in our focus group participants showed a limited insight into routine care practice, which should be complemented by further assessments.

**Trial Registration:** This project has received funding from the European Union’s Horizon 2020 research and innovation programme under grant agreement No. 965328.

**Consent to publish**

Non applicable

**References**

1. Nevedal AL, Reardon CM, Opra Widerquist MA, Jackson GL, Cutrona SL, White BS, et al. Rapid versus traditional qualitative analysis using the Consolidated Framework for Implementation Research (CFIR). Implementation Science. 2021 Jul 2;16(1).

### P33 Implementing organised colorectal cancer screening programs in a decentralised political system - the case of Switzerland

#### Bianca Albers^1^, Reto Auer^2^, Emanuela Nyantakyi^1^, Ekaterina Plys^3^, Clara Podmore^3^, Franziska Riegel^1^, Marie-Therese Schultes^1^, Kevin Selby^3^, Joel Walder^1^, Lauren Clack^1^

##### ^1^Institute for Implementation Science in Health Care (IfIS), University of Zurich, Zurich, Switzerland; ^2^Institute of primary health care (BIHAM), University of Bern, Bern, Switzerland; ^3^Center for primary care and public health (Unisanté), University of Lausanne, Switzerland

###### **Correspondence:** Bianca Albers (bianca.albers@uzh.ch)

*Implementation Science 2023*, **18(Suppl 1):**P33

**Background**

In Switzerland, the early detection of colorectal cancer (CRC) has become a priority on cantonal health policy agendas. In 2022, approximately half of the country’s 26 cantons had established or were preparing an organised CRC screening program. Through these programs, CRC screening is offered systematically to an entire segment of the residents of a canton, using routine stool tests and/or colonoscopy. Since most organised screening programs in Europe were established in the past ten years, there is limited knowledge about how to best implement and sustain them [1, 2]. The aim of this study is to understand current practices in implementing Swiss CRC screening programs and to inform their further development.

**Method**

A mixed methods multiple case study design was developed, including the use of an adapted Implementation Mapping approach (IMA) [3] and the conduct of an integrative systematic literature review [4]. In phase 1, representatives for all established/planned CRC screening programs were interviewed to explore the key characteristics of program implementation. In phase 2 (ongoing), the implementation of four programs will be examined in detail, based on the IMA and additional key stakeholder interviews, and focus groups. While implementation mapping is generally conceptualised as a tool to prospectively guide implementation, the adapted IMA was developed for use with existing implementation practice [3].

**Results**

A unique overview of key program implementation characteristics was generated, reflecting the challenges that emerge from CRC program implementation within the highly decentralised political structure of Switzerland. These and additional results to be gathered during phase 2 will be presented, including experience with the use of the adapted IMA.

**Conclusion**

This study will contribute to the still scarce knowledge base on implementing organised CRC screening programs and will be of relevance to key decision makers initiating, establishing, and maintaining these programs in Switzerland and beyond.

**Literature Review Registration:** The literature review included in this study was registered on PROSPERO CRD42022306580.

**Trial Registration:** Non applicable

**Consent to publish**

Non applicable

**References**

1. Schliemann D, Ramanathan K, Matovu N, O’Neill C, Kee F, Su TT, Donnelly M (2021) The implementation of colorectal cancer screening interventions in low-and middle-income countries: a scoping review. Bmc Cancer 21:1125
2. Priaulx J, Turnbull E, Heijnsdijk E, Csanádi M, Senore C, Koning HJ de, McKee M (2020) The influence of health systems on breast, cervical and colorectal cancer screening: an overview of systematic reviews using health systems and implementation research frameworks. J Health Serv Res Po 25:49–58
3. Schultes M-T, Albers B, Caci L, Nyantakyi E, Clack L (2022) A modified implementation mapping methodology for evaluating and learning from existing implementation. frontiers in Public Health. https://doi.org/10.3389/fpubh.2022.836552
4. Whittemore R, Knafl K (2005) The integrative review: updated methodology. Methodological Issues in Nursing Research 52:546–553

### O34 Using a modified Delphi process to develop a programme theory and inform programme transformation

#### Christina Kien, Viktoria Titscher

##### Department for Evidence-based Medicine and Evaluation, University of Continuing Education, Krems, 3500, Austria

###### **Correspondence:** Christina Kien (christina.kien@donau-uni.ac.at)

*Implementation Science 2023*, **18(Suppl 1):**O34

**Background**

Programme theory can guide evaluation of programmes and streamline the implementation of programmes by different providers. We aimed at developing and applying a systematic process to elucidate and foster a common understanding of the programme theory and focussing on the functions of core elements involving relevant stakeholders of the programme. The health promotion programme follows the WHO health promoting school approach guidelines and aims at enabling representatives of different schools to create a healthy school environment.

**Method**

We conducted seven qualitative interviews with relevant stakeholders (i.e., programme’s providers and lead). Furthermore, we interviewed twelve middle school teachers being responsible for the implementation of the programme in their schools. Two researchers analysed the results applying a thematic analysis [1]. We focused especially on the definition of the functions of the core elements of the programme. These core elements’ functions were then used in a Delphi process involving the same stakeholders. The Delphi process involved four different steps: 1) presentation and clarification of the functions, 2) rating of the functions’ relevance, 3) second rating of the functions’ relevance based on the results of the first rating, and 4) discussion and clarification of remaining functions with stakeholders.

**Results**

Overall, the modified Delphi process enabled to identify 40 relevant out of 107 defined functions for 14 core elements of the programme.

**Conclusion**

This process enabled a fruitful discussion between the programme’s providers and lead about the programme theory. Furthermore, it sharpened the programme theory by focusing on core elements and their most relevant functions. Based on these results, the programme theory was finalised. This process highlighted the necessity of changing our approach to develop a programme theory considering the difficulties of the development from an already existing programme with different providers. Furthermore, this process supported the planning of the evaluation.

**Trial Registration:** Non applicable

**Consent to publish**

Non applicable

**References**

1. Braun V, Clarke V. Using thematic analysis in psychology. Qual Res Psychol. 2006; 3: 77-101.

### O35 A facilitation intervention to increase uptake of an adverse drug event prevention intervention: ActionADE

#### Erica Lau^1,2^, Serena Small^1,2^, Kate Butcher^1,3^, Ellen Balka^1,4^, Corinne Hohl^1,2,5^

##### ^1^Centre for Clinical Epidemiology and Evaluation, Vancouver Coastal Health Research Institute, Vancouver, BC, Canada; ^2^ Department of Emergency Medicine, University of British Columbia, Vancouver, BC, Canada; ^3^ Vancouver General Hospital Pharmacy Department, Vancouver, BC, Canada; ^4^ School of Communication, Simon Fraser University, Burnaby, BC, Canada; ^5^ Vancouver General Hospital Emergency Department, Vancouver, BC, Canada

###### **Correspondence:** Erica Lau (erica.lau@ubc.ca)

*Implementation Science 2023*, **18(Suppl 1):**O35

**Background**

Adverse drug events (ADE) are a leading cause of emergency department visits and hospital admissions in Canada [1,2]. ActionADE aims to prevent repeat ADE by enabling clinicians to document and communicate standardized ADE information across care settings. We describe a 5-month facilitation intervention to promote uptake of ActionADE in four hospitals in British Columbia, Canada.

**Method**

In this multiple case study, we used a four-step iterative facilitation process [3]: i) conduct formative evaluation to identify barriers to use, ii) generate site-specific implementation plan using the Consolidation framework for implementation research-Expert Recommendations for Implementing Change (CFIR-ERIC) implementation strategy matching tool [4], iii) co-create functions and forms [5] of the implementation strategies with site champions, and iv) execute, monitor process and evaluate outcomes. Implementation outcomes included the number and types of implementation strategies, changes in the number of monthly ADE reports and active users before (Jun to Oct 2021) and after (Nov 2021 to Mar 2022) the facilitation process.

**Results**

Through the facilitation process, we identified four functions (create tension for change, support integration, provide access to intervention information and increase clinician’s awareness, knowledge and skills) and 4 to 8 corresponding forms for each site (e.g., engage and prepare additional champions, 1-on-1 follow-ups). Sites’ responses to the facilitation process varied. The number of monthly ADE reports increased substantially in sites A (+700%) and B (+84%) and declined in sites C (-29%) and D (-8%). The number of active users increased in site A (+47%) and D (+68%) and declined in sites B (-7%) and C (-23%). Contextual factors that influenced the facilitation process (e.g., staff shortage, roles of champions) also varied by site.

**Conclusion**

This study illustrates a systematic process for researchers and stakeholders to prospectively co-create core functions and forms of implementation interventions according to local contexts’ characteristics.

**Trial Registration:** Non applicable

**Consent to publish**

Non applicable

**References**

1. Zed PJ, Abu-Laban RB, Balen RM, Loewen PS, Hohl CM, Brubacher JR, et al. Incidence, severity and preventability of medication-related visits to the emergency department: a prospective study. CMAJ. 2008;178(12):1563-9.
2. Maity TS, Longo CJ. Adverse Drug Reactions in Canada (2009-2018): Insights from the Canada Vigilance Database. Healthcare quarterly (Toronto, Ont). 2020;23(1):40-6.
3. Gustavson AM, Wisdom JP, Kenny ME, Salameh HA, Ackland PE, Clothier B, et al. Early impacts of a multi-faceted implementation strategy to increase use of medication treatments for opioid use disorder in the Veterans Health Administration. Implementation Science Communications. 2021;2(1):20.
4. Waltz TJ, Powell BJ, Fernández ME, Abadie B, Damschroder LJ. Choosing implementation strategies to address contextual barriers: diversity in recommendations and future directions. Implementation Science. 2019;14(1):42.
5. Kirk MA, Haines ER, Rokoske FS, Powell BJ, Weinberger M, Hanson LC, et al. A case study of a theory-based method for identifying and reporting core functions and forms of evidence-based interventions. Translational behavioral medicine. 2021;11(1):21-33.

### P36 Development and implementation of educational prescribing resources to mental health pharmacists to improve the physical health of people with severe mental illness

#### Annabel Lane^1,^ Sofia Dewji^1^, Karen Ang^1,2^, Siobhan Gee^1,2,4^, Raymond McGrath^1,2^, Rina Patel^4^, Nick Sevdalis^3^, Julie Williams^3^ on behalf of the IMPHS study group

##### ^1^South London and Maudsley NHS Foundation Trust, London, UK; ^2^Mind and Body Programme, King’s Health Partners, Guy’s Hospital, London, UK; ^3^Centre for Implementation Science, King’s College London, London, UK; ^4^King's College Hospital NHS Foundation Trust, London, UK

###### **Correspondence:** Annabel Lane (Annabel.lane@slam.nhs.uk)

*Implementation Science 2023*, **18(Suppl 1):**P36

**Background**

People with serious mental illnesses (SMI) live on average 15-20 years less than the general population, partly due to physical health comorbidities. Improving the physical health knowledge of mental health pharmacists could assist in reducing the mortality gap in people with SMI. We describe the development and implementation of educational materials for mental health pharmacists at a large UK mental health trust.

**Method**

Physical health training needs were identified using a survey with pharmacists. We implemented (1) monthly, educational webinars covering different physical health topics, and (2) specific physical health guidelines, circulated to all mental health trust pharmacists. Questionnaires and interviews were undertaken with pharmacists to evaluate impact and implementation.

**Results**

106 individual staff attended the webinars. Common themes from the questionnaire (n=15) and interviews (n=8) were that the webinars were ‘good-refreshers’, concise and provided appropriate level, pharmacy specific information. Common barriers for webinar attendance were high workload and other work commitments. 50% of the interviewed pharmacists were not aware of the guidelines and only two pharmacists had read them. The implementation evaluation further revealed that the co-design approach with pharmacists enabled interprofessional relationships (i.e. acute and mental health pharmacists) and tailoring of educational content. Trust-wide pharmacy leadership buy-in and administrative support also boosted implementation.

**Conclusion**

These barriers reflect the challenges of developing interventions in a pressurised hospital setting. To overcome these barriers, co-designing with expert pharmacists is key. Regular meetings, establishing role clarity and accountability, building a relationship with the acute hospital pharmacy team, and dedicated funding enabled this.

Continual education for clinicians is key to ensuring service users experience the best available care including physical healthcare expertise. To sustain the interventions, dedicated administrative and leadership resource is required to establish accountability and responsibility. We also identified the need to publicise implementations and improve access to resources.

**Trial Registration:** Non applicable

**Consent to publish**

SLaM clinical governance and Information governance approvals

### P37 Beyond input-output: Applying dynamic systems theory to the complexity of implementing mental health interventions in non-western cultures

#### Adele Pacini^1,2^ Prithvi Shrestha^3^

##### ^1^School of Psychology and Counselling, Faculty of Arts and Social Sciences, The Open University, Milton Keynes, UK; ^2^The Gatehouse Charity, Bury St Edmunds, UK; ^3^School of Languages and Applied Linguistics, Faculty of Wellbeing, Education and Languages, The Open University, Milton Keynes, UK

###### **Correspondence:** Adele Pacini (a.pacini@open.ac.uk)

*Implementation Science 2023*, **18(Suppl 1):**P37

**Background**

Global dissemination of western mental health interventions across widely diverse cultures leaves a potentially large implementation gap for non-Caucasian people [1]. Central to the uptake of mental health interventions are the extent to which they align with the cultural and personal values of local cultures, organisations, staff, individuals and their families.

**Method**

We explore the potential of integrating components of the Pragmatic Robust Implementation and Sustainability Model (PRISM) [2] within a dynamic system of cultural adaptation [3]. We model the complementary and contradictory perspectives on cultural concepts of distress and healing, highlighting how successful implementation depends on navigating the ‘best fit’ between these concepts and evidence based psychological techniques.

**Results**

Figure 1 shows the resulting model for the implementation of culturally adapted psychological interventions. We model the dynamic nature of the overlap between an individual’s coping mechanisms, their family’s, alongside organizational capacity to implement interventions and existing cultural and evidence-based practices to support mental health.

**Conclusion**

Integrating components of the PRISM within a dynamic system model of cultural adaptation allows us to represent the uncertainty and unpredictability of adapting mental health interventions more accurately in non-western cultures. Importantly, it also models the tension between self, other and organizational values, which may be particularly critical in collectivist cultures, or across generations in countries experiencing rapid development. Our case example suggests how we might navigate these uncertainties and complexities through a lens of ‘best fit’ rather than input-output.

**Trial Registration:** Non applicable

**Consent to publish**

Non applicable

**References**

1. Rose, D., Kalathil, J. Power, privilege and knowledge: the untenable promise of co-production in mental “health”. *Frontiers in Sociology*. 2019;57. Available from; doi.org/10.33389/fsoc.2019.00057
2. Feldstein, A.C., Glasgow, R.E. A practical, robust implementation and sustainability model (PRISM) for integrating research findings into practice. *The Joint Commission Journal on Quality and Patient Safety*. 2008;34(4):228-43. Available from; https://doi.org/10.1016/S1553-7250(08)34030-6
3. Braithwaite, J., Churruca, K., Long, J.C., Ellis, L.A. Herkes, J. When complexity science meets implementation science: a theoretical and empirical analysis of systems change. *BMC medicine*. 2018;16(63):1-4. Available from; doi.org/10.1186/s12916-018-1057-z

**Fig. 1 (abstract P37).**
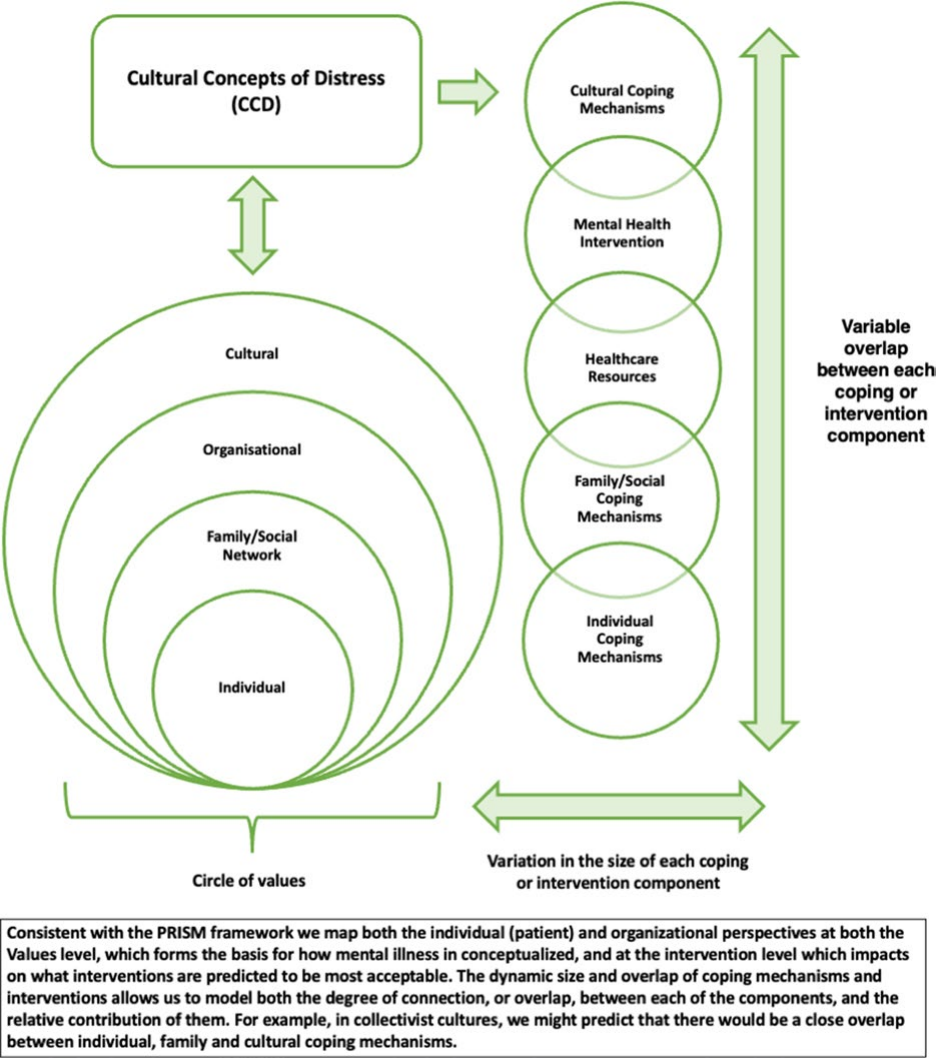
Combining the PRISM with dynamic mental health coping and intervention components

### O38 Challenges to implementing person-centred outcome measures into routine paediatric palliative care

#### Hannah M Scott^1^, Lucy Coombes^1,2^, Debbie Braybrook^1^, Daney Harðardóttir^1^, Anna Roach^1^, Katherine Bristowe^1^, Clare Ellis-Smith^1^, Richard Harding^1^, on behalf of C-POS

##### ^1^Florence Nightingale Faculty of Nursing Midwifery and Palliative Care, Cicely Saunders Institute, King’s College London, London, UK; ^2^Royal Marsden NHS Foundation Trust, London, UK

###### **Correspondence:** Hannah M Scott (hannah.m.scott@kcl.ac.uk)

*Implementation Science 2023*, **18(Suppl 1):**O38

**Background**

To successfully implement a newly developed measure into clinical practice, the challenges to implementation must be understood [1]. Previous research has focused on disease-specific or generic Quality of Life measures in paediatric healthcare, or the use of outcome measures in adult palliative care [2-4]. Evidence identifying the perspectives of all key stakeholder groups is needed to ensure successful implementation of new person-centred outcome measures (PCOMs) in the paediatric palliative care context.

**Method**

Semi-structured interviews with purposively sampled key stakeholders. Children with life-limiting or life-threatening conditions (LLLTC), parents/carers and siblings of children with LLLTC, and health and social care professionals (HSCPs) caring for children with LLLTC were recruited from 9 UK sites. Commissioners of UK paediatric palliative care services were recruited via a non-governmental organisation or direct recommendations. Verbatim transcripts were analysed using a Framework approach analysis and inductive coding in NVivo.

**Results**

103 interviews were conducted with 106 participants (26 children, 40 parents/carers, 13 siblings, 15 HSCPs, and 12 commissioners). Potential challenges identified by HSCP and commissioners included: (1) gatekeeping by family members and (2) added workload for already stretched services. Potential challenges identified by children included: (1) trusting who administered the measure and (2) privacy concerns around who could access the results. Family members also identified potential challenges relating to (1) added workload for HSCP and (2) privacy concerns around who could access the results.

**Conclusion**

Whilst some challenges were identified as concerns across multiple stakeholder groups, other challenges identified were unique to specific stakeholder groups. Understanding these different and over lapping perspectives of the perceived challenges is essential for the development of concomitant strategies for implementation of a new PCOM into paediatric healthcare practice. Which in turn helps to support uptake of a PCOM into routine practice.

**Trial Registration:** Non applicable

**Consent to publish**

Non applicable

**References**

1. Greenhalgh J. The applications of PROs in clinical practice: what are they, do they work, and why? Quality of Life Research. 2009;18(1):115-23.
2. Antunes B, Harding R, Higginson IJ. Implementing patient-reported outcome measures in palliative care clinical practice: A systematic review of facilitators and barriers. Palliative Medicine. 2014;28(2):158-75.
3. Anderson LM, Papadakis JL, Vesco AT, Shapiro JB, Feldman MA, Evans MA, et al. Patient-Reported and Parent Proxy-Reported Outcomes in Pediatric Medical Specialty Clinical Settings: A Systematic Review of Implementation. Journal of Pediatric Psychology. 2020;45(3):247-65.
4. Howell D, Molloy S, Wilkinson K, Green E, Orchard K, Wang K, et al. Patient-reported outcomes in routine cancer clinical practice: a scoping review of use, impact on health outcomes, and implementation factors. Annals of Oncology. 2015;26(9):1846-58.

### P39 “Mindfulness for parents who care” or “Mindfulness for parent carers”? Re-framing a mindfulness course to align with parent carer’s identity as a parent before a carer increases uptake: A formative evaluation

#### Gemma Hawkins^1,2^, Annabel Stickland^2^, Adele Pacini^1,2^

##### ^1^School of Psychology and Counselling, Faculty of Arts and Social Sciences, The Open University, Milton Keynes, UK; ^2^The Gatehouse Charity, Bury St Edmunds, UK

###### **Correspondence:** Gemma Hawkins (gfharris@gmail.com)

*Implementation Science 2023*, **18(Suppl 1):**P39

**Background**

Parent carers of children with special educational needs have an increased risk of mental and physical ill-health [1,2]. It remains problematic to engage parent carers in wellbeing support [3], with many parents not perceiving themselves as ‘carers’ [4]. Following low uptake to our Mindfulness for Parent Carers (MPC) group we carried out a formative evaluation and utilised ecological theory aligned with public health goals as outlined by Atkins et al [5]. We examined whether expressions of interest (EOI), and applications to, the MPC group were increased by aligning the promotion of the group with parent carer’s identity and through settings that support that identity.

**Method**

For intake one, the course was promoted as ‘Mindfulness for Parent Carers’ via email, poster and telephone contacts to local carer charities, NHS services, and the voluntary action mailing list (a reach of 1,300 individuals). For intake two the course was promoted as ‘Mindfulness for Parents who Care’ via local workplace settings (18) and primary and secondary schools (397 including 15 special needs schools).

**Results**

For the EOI questionnaire, fourteen people completed the EOI questionnaire for intake one, and seventeen people for intake two. The difference was not significant (c^2^(1) = 0.29 p = 0.59). For full applications, intake one had one application; intake two had six applications. There were significantly more applications made in intake two than intake one c^2^ (1) = 3.57, p = 0.05.

**Conclusion**

Aligning intervention promotional material with both parent carer’s primary identity (ie a parent first), and setting (ie schools/workplace) resulted in a significantly greater number of applications to the MPC group. However, numbers were low across both intakes, and thus more work is needed to understand how to work with parent carers and offer support how and when they need it.

**Trial Registration:** Non applicable

**Consent to publish**

Non applicable

**References**

1. Emerson E. Mothers of children and adolescents with intellectual disability: social and economic situation, mental health status, and the self-assessed social and psychological impact of the child’s difficulties. Journal of Intellectual Disability Research. 2003 May;47(4-5):385–99.
2. Lee MH, Park C, Matthews AK, Hsieh K. Differences in physical health, and health behaviors between family caregivers of children with and without disabilities. Disability and Health Journal. 2017 Oct;10(4):565–70.
3. Moriarty J, Manthorpe J, Cornes M. Reaching out or missing out: approaches to outreach with family carers in social care organisations. Health & Social Care in the Community. 2014 Oct 21;23(1):42–50.
4. O’Connor DL. Self-identifying as a caregiver: Exploring the positioning process. Journal of Aging Studies [Internet]. 2007 Apr 1;21(2):165–74. Available from: https://www.sciencedirect.com/science/article/abs/pii/S089040650600096X
5. Atkins MS, Rusch D, Mehta TG, Lakind D. Future Directions for Dissemination and Implementation Science: Aligning Ecological Theory and Public Health to Close the Research to Practice Gap. Journal of Clinical Child & Adolescent Psychology. 2015 Jul 9;45(2):215–26.

### P40 Implementation of home practice support strategies for older adults attending an online mindfulness based cognitive therapy course: An adaptive intervention protocol

#### Adele Pacini^1,2^, Annabel Stickland^2^, Krystal Iniguez^2^, Gina Di Malta^1^

##### ^1^School of Psychology and Counselling, Faculty of Arts and Social Sciences, The Open University, Milton Keynes, UK; ^2^The Gatehouse Charity, Bury St Edmunds, UK

###### **Correspondence:** Adele Pacini (a.pacini@open.ac.uk)

*Implementation Science 2023*, **18(Suppl 1):**P40

**Background**

Mindfulness based cognitive therapy (MBCT) is a NICE recommended treatment for recurrent depression [1]. Daily home practice is an essential part of MBCT to promote clinical change [2,3], but is inconsistently completed our course participants. Using self-report data from an online MBCT course for older adults we describe a formative evaluation to develop an adaptive intervention protocol with the aim of improving home practice compliance.

**Method**

Participants (n = 55) attended an online MBCT course and were issued with audio files from which to practice daily between sessions. Weekly questionnaires were completed where participants recorded frequency of practice (x̄ = 3.68, SD = 1.69 days) and what impacted on their ability to complete daily practice. Focus groups were held after each course (four in total) and thematic analysis identified successful strategies alongside challenges faced with home practice.

**Results**

The behaviour change wheel (BCW) [4] was used to map out participant’s experiences of home practice. Figure 1 shows the resultant framework, with quotes from participants illustrating key themes from the focus groups. The strategies to be used in the second arm of our adaptive intervention are shown in the ‘intervention functions’ column.

**Conclusion**

The BCW provided a good fit for mapping participant’s experiences of home practice. Quantitative and qualitative data on home practice from the second arm of the adaptive intervention will be used to evaluate the feasibility and acceptability of proposed strategies.

**Trial Registration:** Non applicable

**Consent to publish**

Non applicable

**References**

1. National Institute for Health and Care Excellence (NICE). Depression in adults: treatment and management [Internet]. [London]: NICE; 2022. Available from: https://www.nice.org.uk/guidance/ng222
2. Kabat-Zinn J. Full Catastrophe Living: Using the Wisdom of Your Body and Mind to Face Stress, Pain and Illness. New York United States: Dell Publishing; 1990.
3. Vettese LC, Toneatto T, Stea JN, Nguyen L, Wang JJ. Do Mindfulness Meditation Participants Do Their Homework? And Does It Make a Difference? A Review of the Empirical Evidence. Journal of Cognitive Psychotherapy. 2009 Aug;23(3):198–225.
4. Michie S, van Stralen MM, West R. The Behaviour Change wheel: a New Method for Characterising and Designing Behaviour Change Interventions. Implementation Science. 2011 Apr 23;6(1).

**Fig. 1 (abstract P40).**
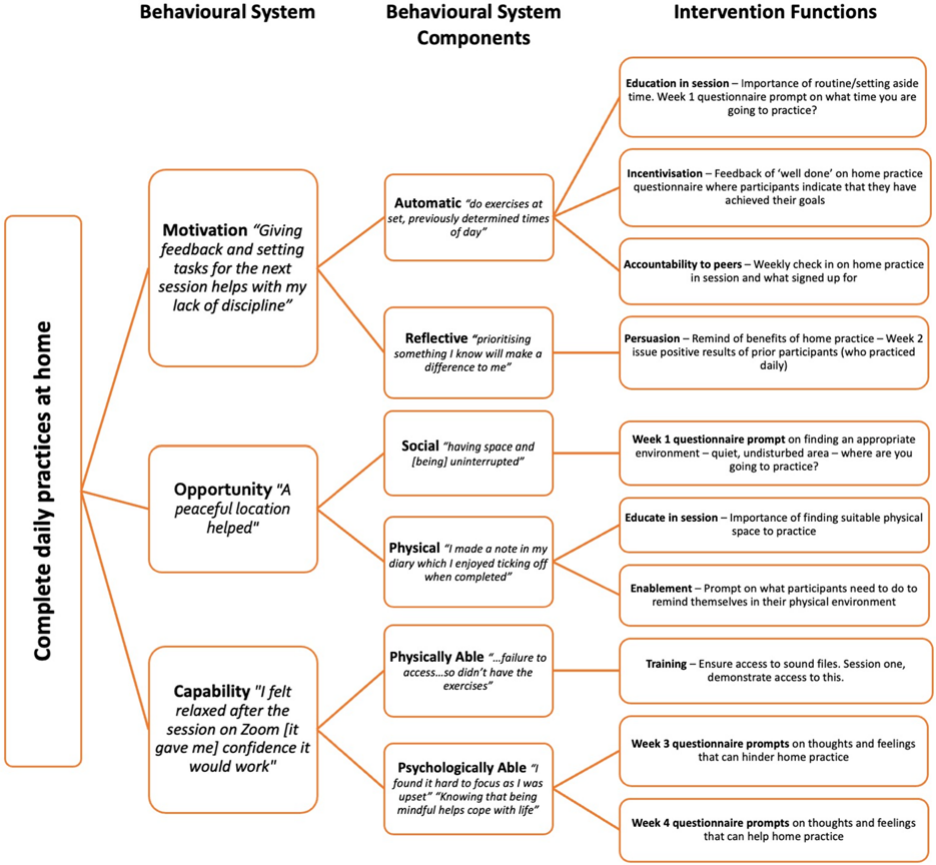
A Participant’s experiences of home practice applied to the BCW alongside adaptions to the intervention

### O41 Development of implementation strategies to overcome barriers when implementing a combined lifestyle intervention for community-dwelling older people in community-care settings

#### Patricia J van der Laag^1^, Berber G Dorhout^2,3^, Aaron A Heeren^1^, Di-Janne JA Barten^2,4^, Cindy Veenhof^2,4^, Lisette Schoonhoven^1,5^

##### ^1^Julius Center for Health Sciences and Primary Care, Nursing Science, University Medical Center Utrecht, University Utrecht, Utrecht, The Netherlands; ^2^Research Group Innovation of Human Movement Care, Research Centre for Healthy and Sustainable Living, Utrecht University of Applied Sciences, Utrecht, The Netherlands; ^3^Division of Human Nutrition and Health, Wageningen University and Research, The Netherlands; ^4^Department of Rehabilitation, Physical Therapy Science & Sports, University Medical Center Utrecht, Utrecht University, Utrecht, The Netherlands; ^5^Faculty of Health Sciences, University of Southampton, UK

###### **Correspondence:** Patricia J van der Laag (P.j.vanderlaag-3@umcutrecht.nl)

*Implementation Science 2023*, **18(Suppl 1):**O41

**Background**

ProMuscle is a combined lifestyle intervention that has shown to be effective in improving muscle mass, muscle strength, and physical functioning in community-dwelling older adults. Potentially, it could facilitate older people in maintaining their functional independence.

To increase the likelihood of successful implementation of ProMuscle, this study aims to develop appropriate implementation strategies targeting previously identified barriers to implement ProMuscle in community-care.

**Method**

A theory-informed approach was adopted to develop appropriate implementation strategies, consisting of four subsequent steps. First, previously identified barriers for implementation were categorized into the constructs of the Consolidated Framework for Implementation Research (CFIR) [1], including the underlying theoretical constructs. Second, the CFIR-ERIC matching Tool linked barriers to implementation strategies. Behavioral change strategies were added from literature. Third, evidence for implementation strategies was sought in literature. Fourth, in co-creation with involved healthcare professionals and implementation experts, implementation strategies were operationalized to practical implementation activities following the guidance of Proctor. Lastly, an implementation plan that can be tailored to individuals’ context was developed, prioritizing implementation activities over time.

**Results**

A total of 654 barriers were categorized to the CFIR framework. The majority of barriers were related to the CFIR domain outer setting. Subsequently, the identified barriers were linked to 37 unique strategies. As many strategies affected multiple barriers, strategies were assigned in eight overarching themes: assessing the context, network internally, network externally, costs, education, process, champions, content of the intervention, and behavioral change of the end-users.

Co-creation sessions with professionals and implementation-experts resulted in tangible implementation actions, processed into an online implementation toolbox that supports healthcare professionals chronologically during the implementation process.

**Conclusion**

The theory-informed approach in combination with co-creation led to the development of practical multicomponent implementation strategies to implement ProMuscle. Next step is to evaluate the implementation strategies including the implementation toolbox regarding the implementation of ProMuscle in community-care.

**Trial Registration:** Non applicable

**Consent to publish**

Non applicable

**References**

1. Damschroder LJ, Aron DC, Keith RE, Kirsh SR, Alexander JA, Lowery JC. Fostering implementation of health services research findings into practice: A consolidated framework for advancing implementation science. Implement Sci. 2009;4(1).

### O42 Experiences and perceptions of evidence use among senior health service stakeholders: A qualitative study

#### Susan Calnan, Sheena McHugh

##### School of Public Health, University College Cork, Ireland

###### **Correspondence:** Susan Calnan (susan.calnan@ucc.ie)

*Implementation Science 2023*, **18(Suppl 1):**O42

**Background**

The importance of using robust evidence to inform policy and decision-making in health is widely acknowledged. Nevertheless, the evidence-to-policy and practice gap continues to persist. The aim of this study was to: examine senior health service stakeholders’ experiences and perceptions of evidence us; identify barriers to and facilitators of research use; and identify recommendations to support research use among health service stakeholders.

**Method**

A qualitative study was undertaken using semi-structured one-to-one interviews with a sample of senior health service stakeholders in Ireland. Interviews were conducted in late August 2021 to January 2022, and ethical approval for the study was granted by the university ethics committee. Purposive sampling was used, and inclusion criteria were national-level senior management involved in making decisions regarding strategy, planning, development and delivery of health services. Interviews were analysed using thematic analysis.

**Results**

A total of 17 interviews were conducted (response rate 38%). Participants reported using a range and mix of evidence types to inform their work and decision-making, and they had a strong appreciation of the importance of research. Key barriers to research use included lack of time, relevance and quality of the research, organisational culture, and other stakeholders’ lack of understanding or interest in research. Key facilitators included the organisation’s library service, activities to improve the dissemination of research findings, and links with universities.

**Conclusion**

The study concludes that health service stakeholders have a broad conceptualisation of evidence, viewing research as one type of evidence and recognising the value of evidence in informing work and decision-making. Despite this, the study underlines key areas for improvement, including the need for a more strategic approach to research and for more resources to facilitate research use. Knowledge translation strategies have the potential to facilitate greater research use in the organisation, defined according to ‘push’, ‘pull’ and ‘exchange’ efforts.

**Trial Registration:** Non applicable

**Consent to publish**

Non applicable

### O43 Understanding How Approaches to Implementation Support Have Evolved Over Time to Advance Improved and Equitable Outcomes in Human Service Systems

#### Allison Metz^1^, Todd Jensen^1^, Amanda Farley^1^, Annette Boaz^2^

##### ^1^University of North Carolina at Chapel Hill; ^2^London School of Hygiene and Tropical Medicine

###### **Correspondence:** Allison Metz (allison.metz@unc.edu)

*Implementation Science 2023*, **18(Suppl 1):**O43

**Background**

Implementation support has become a frequently used approach to strengthen organizational efforts to sustainably use evidence. In utilizing implementation support, agencies and funders collaborate with implementation support practitioners (ISPs) whose explicit role it is to support the implementation of evidence-informed practices [1-3]. The goals of this study were to understand what experienced ISPs have learned about supporting evidence use in service systems, and how their approach to providing implementation support has shifted over time as a result of this learning.

**Method**

A purposive sample of 17 experienced ISPs participated in in-depth interviews. A semi-structured interview guide was used to ascertain participants’ perceptions about various aspects of their work providing implementation support. Data were analyzed using a narrative analysis approach, focusing on broad elements that highlighted the trajectory of respondents’ professional journey in the context of providing implementation support. A team engaged in data coding and analysis in an effort to triangulate observations and maintain consensus with respect to emerging findings.

**Results**

Respondents foregrounded the development of five main components to their approach in supporting evidence use: (a) supporting participatory learning; (b) engaging in co-creation; (c) building trusting relationships; (d) understanding context and community perspectives; and (e) supporting communication, coordination and collaboration. Interviewees described a necessary evolution in their approach to supporting evidence use. Three main shifts in implementation support practice were observed: (a) didactic to participatory approaches, (b) expert-driven to co-creation approaches, and (c) framework-based to relationship-focused approaches

**Conclusion**

Respondents highlighted the need to move away from top-down approaches towards a model of multi-level support focused on co-creation, peer learning, and collaborative work. At the heart of this work is development of trusting relationships. All interviewees reported that high quality relationships between ISPs and stakeholders was the most critical factor for achieving implementation results.

**Trial Registration:** Non applicable

**Consent to publish**

Non applicable

**References**

1. Albers B, Metz A, Burke K, Bührmann L, Bartley L, Driessen P, et al. Implementation Support Skills: Findings From a Systematic Integrative Review. Research on Social Work Practice. 2020 Oct 27;31(2):147–70.
2. Metz A, Albers B, Burke K, Bartley L, Louison L, Ward C, et al. Implementation Practice in Human Service Systems: Understanding the Principles and Competencies of Professionals Who Support Implementation. Human Service Organizations: Management, Leadership & Governance. 2021 Mar 15;45(3):1–22.
3. Albers B, Metz A, Burke K, Bührmann L, Bartley L, Driessen P, et al. The Mechanisms of Implementation Support - Findings from a Systematic Integrative Review. Research on Social Work Practice. 2021 Nov 23;32(3):259–80.

### O44 Competencies for supporting evidence use: The role of trusting relationships in implementation

#### Allison Metz^1^, Todd Jensen^1^, Amanda Farley^1^, Annette Boaz^2^

##### ^1^University of North Carolina at Chapel Hill; ^2^London School of Hygiene and Tropical Medicine

###### **Correspondence:** Allison Metz (allison.metz@unc.edu)

*Implementation Science 2023*, **18(Suppl 1):**O44

**Background**

There is an increasing call for the advancement of a workforce capable of integrating implementation research – models, frameworks, and strategies – into practice to support evidence use, advance equity, and achieve improved population outcomes. Studies have identified plausible competencies for implementation practice [1-3]. This William T. Grant funded study explored the use of competencies by professionals who support evidence use in human service systems and the conditions under which specific implementation strategies were perceived as most effective.

**Method**

A hybrid purposive-convenience sampling approach resulted in a sample of 17 individuals, each with more than 15 years’ experience providing implementation support. Data were collected via in-depth, semi-structured interviews. Core research questions included: What implementation support strategies are used to support the use of evidence? Under what conditions have specific implementation support strategies contributed to supporting evidence use? Data were analyzed using a qualitative content analysis approach.

**Results**

Respondents reported using a range of strategies across domains to support evidence-use. Trusting relationships emerged as a ubiquitous fixture of the implementation support process. Respondents described trusting relationships as directly associated with successful implementation and use of evidence and bidirectionally associated with (and reinforcing of) all other implementation strategies.

**Conclusion**

Findings reflect that implementation support is a multi-faceted endeavor that requires a broad range of skills. Respondents enacted technical strategies (e.g., frequent interactions), while simultaneously carrying out relational strategies (e.g., empathy-driven exchanges). Relationships appear to be as important as technical strategies and may explain why perfectly offered implementation support at times remains unsuccessful in leading to sustained evidence use. Building a workforce capable of supporting evidence-use will require developing skills for building trusting relationships. Findings from this study have resulted in a model for trust building being tested by NJ’s Division of Children and Families with funding from the W.T. Grant Foundation.

**Trial Registration:** Non applicable

**Consent to publish**

Non applicable

**References**

1. Albers B, Metz A, Burke K, Bührmann L, Bartley L, Driessen P, et al. Implementation Support Skills: Findings From a Systematic Integrative Review. Research on Social Work Practice. 2020 Oct 27;31(2):147–70.
2. Metz A, Albers B, Burke K, Bartley L, Louison L, Ward C, et al. Implementation Practice in Human Service Systems: Understanding the Principles and Competencies of Professionals Who Support Implementation. Human Service Organizations: Management, Leadership & Governance. 2021 Mar 15;45(3):1–22.
3. Albers B, Metz A, Burke K, Bührmann L, Bartley L, Driessen P, et al. The Mechanisms of Implementation Support - Findings from a Systematic Integrative Review. Research on Social Work Practice. 2021 Nov 23;32(3):259–80.

### O45 Developing an initial programme theory of prehospital feedback in an ambulance service setting: A mixed-methods study

#### Caitlin Wilson^1,2^, Dr Gillian Janes^3^, Prof Rebecca Lawton^4^, Dr Jonathan Benn^1,4^

##### ^1^School of Psychology, University of Leeds, Leeds, UK; ^2^North West Ambulance Service NHS Trust, Bolton, Lancashire, UKK; ^3^Faculty of Health, Psychology and Social Care, Manchester Metropolitan University, Manchester, UKK; ^4^Bradford Institute for Health Research, Bradford Teaching Hospitals NHS Foundation Trust, Bradford, UK

###### **Correspondence:** Caitlin Wilson (hc15c2w@leeds.ac.uk)

*Implementation Science 2023*, **18(Suppl 1):**O45

**Background**

Evidence exists for the effectiveness of feedback in changing professional behaviour and improving clinical performance across a range of healthcare settings, but this has not yet been explored within the prehospital context [1]. The aim of this study was to understand how UK ambulance services are meeting the challenge of providing feedback and generate an initial explanatory programme theory to capture the implicit mechanisms by which prehospital feedback results in desirable outcomes.

**Method**

This mixed methods study combines a realist evaluation framework with an explanatory case study design. The study consisted of a national cross-sectional survey to identify active and historic feedback initiatives in UK ambulance services, followed by 4 in-depth case studies of these initiatives. Case studies were purposively selected from survey responses using a sampling framework stratified by feedback type and context, and each involved 4-5 semi-structured qualitative interviews and documentary analysis.

An initial programme theory was developed using the survey data and findings from our previously conducted systematic review and exploratory interview study. It was informed by existing theories on audit and feedback, behaviour change and implementation science: Clinical Performance Feedback Intervention Theory [2], Theoretical Domains Framework [3] and Implementation Outcomes Evaluation Framework [4].

**Results**

Fitting the descriptive survey data of prehospital feedback initiatives to the CMO framework gave rise to an initial programme theory for prehospital feedback, which is depicted visually in a logic model (Figure 1).

**Conclusion**

Our initial programme theory will be further refined during the ongoing case study phase of this study.

**Trial Registration:** Non applicable

**Consent to publish**

Non applicable

**References**

1. Ivers N, Jamtvedt G, Flottorp S, Young JM, Odgaard-Jensen J, French SD, et al. Audit and feedback: effects on professional practice and healthcare outcomes. Cochrane Database of Systematic Reviews. 2012(6).
2. Brown B, Gude WT, Blakeman T, van der Veer SN, Ivers N, Francis JJ, et al. Clinical Performance Feedback Intervention Theory (CP-FIT): a new theory for designing, implementing, and evaluating feedback in health care based on a systematic review and meta-synthesis of qualitative research. Implementation science : IS. 2019;14(1):40.
3. Michie S, Johnston M, Abraham C, Lawton R, Parker D, Walker A. Making psychological theory useful for implementing evidence based practice: a consensus approach. Quality & safety in health care. 2005;14(1):26-33.
4. Proctor E, Silmere H, Raghavan R, Hovmand P, Aarons G, Bunger A, et al. Outcomes for Implementation Research: Conceptual Distinctions, Measurement Challenges, and Research Agenda. Administration and Policy in Mental Health and Mental Health Services Research. 2011;38(2):65-76.

**Fig. 1 (abstract O45).**
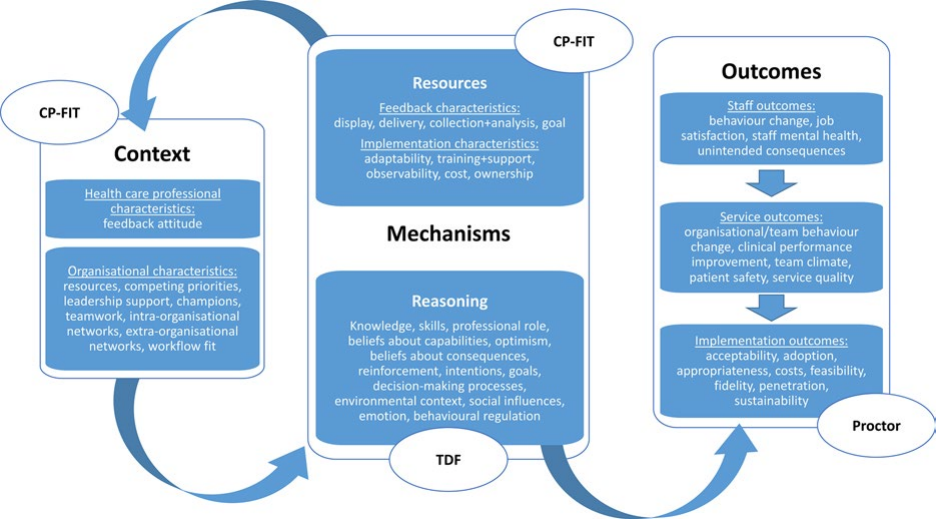
Logic Model of an Initial Programme Theory of Prehospital Feedback

### P46 2 Young Lives: a pilot hybrid type 2 trial of a mentoring scheme for pregnant adolescent girls in Sierra Leone

#### Cristina Fernandez Turienzo^1**†**^, Mangenda Kamara^2**†**^, Lucy November^1**†**^, Prince T Williams^3^, Philmenon Kamara^3^, Suzanne Thomas^2^, Venetia Goodhart^2^, Alex Ridout^1^, Betty Sam^2^; Paul T Seed^1^, Jane Sandall^1**††**^, Andrew H Shennan^1**††**^, on behalf of NIHR CRIBS Group

##### ^1^Welbodi Partnership, Freetown, Sierra Leone; ^2^Department of Women and Children’s Health, Faculty of Life Sciences & Medicine, King’s College London, United Kingdom; ^3^Lifeline Nemeniah Projects, Freetown, Sierra Leone

###### **Correspondence:** Cristina Fernandez Turienzo (cristina.fernandez_turienzo@kcl.ac.uk)

*Implementation Science 2023*, **18(Suppl 1):**P46

^**†**^Joint first authors

^**††**^Joint senior authors

**Background**

Sierra Leone (SL) has one of the highest maternal mortality in the world. Adolescent girls are particularly vulnerable, many times belong to disadvantaged communities usually driven by poverty, lack of education and employment opportunities [1]. In 2015, a household survey conducted in Kuntorloh (Wellington) showed a maternal mortality of 1 in 10 among under 18 years old [2]. A year later, a qualitative study exploring causes of adolescent maternal mortality in this population [2] found important factors in relation to a) vulnerability to adolescent pregnancy (i.e., not living with birth family, sex for water/grades/school fees, criminal justice system, availability & accessibility of contraception and abortion) and vulnerability to death when pregnant (i.e. neglect, abandonment; being cared for by a non-parental adult, delayed care seeking, obstetric risks/socio-economic factors). Cross-cutting factors: Gendered social norms for sexual behaviour. A mentoring scheme for pregnant girls was locally developed and started in October 2017 (5 more teams up to Mar 2021) with promising results [3]. Further funding for a pilot trial was obtained as part of a NIHR Global Health Research Group (CRIBS) that started in Sep 2021 and aims to develop, implement simple, scalable innovations to reduce maternal and perinatal mortality in Sierra Leone [4].

**Method**

We aim to assess the feasibility and implementation of the 2YL mentorship scheme for adolescent pregnant girls in new communities to inform trial procedures for a subsequent fully powered cluster RCT. We are conducting a hybrid type 2, parallel-group pilot cluster RCT in communities served by 12 PHUs covering rural + urban areas. The primary clinical outcome is a composite of maternal and neonatal mortality. We will conduct a nested evaluation of the implementation, mechanisms, and experiences of care, health and wellbeing using mixed methods (e.g. focus groups, semi-structured interviews with adolescents, mentors, PHU staff, community members, friends/relatives; photovoice).

**Results**

The project is ongoing and we highlighted below overall progress so far:

- 2YLs started as part of NIHR CRIBS on Sept 2021
- Research staff recruited & trained
- Project materials developed, ethics approvals obtained, online database developed.
- Local PhD studentship awarded
- Cluster randomisation and community engagement activities in cluster sites completed
- Recruitment and training of mentors ongoing.
- Mentoring intervention to start in June 2022
- An overview of the plans for the implementation evaluation will be presented.

**Trial Registration:** ISRCTN registry (ISRCTN32414369, prospectively registered, 16 March 2022).

**Acknowledgements:**

We would like to thank all girls, families, mentors, community stakeholders and all members of the CRIBS Group & collaborators.

**Consent to publish**

Non applicable

**References**

1. UNICEF. Maternal, neonatal and child health [Internet]. Available from: [https://www.unicef.org/sierraleone/maternal-neonatal-and-child-health
2. November L, Sandall J. ‘Just because she’s young, it doesn’t mean she has to die’: exploring the contributing factors to high maternal mortality in adolescents in Eastern Freetown; a qualitative study. Reproductive Health. 2018 Dec;15(1):1-8.
3. Kamara M, November L. (2018) 2 Young Lives: a mentoring scheme for pregnant teenagers: A feasibility study; October 2017 to September 2018. Report.
4. National Institute for Health Research. Funding and Awards: NIHR Global Health Research Group: Implementation of simple solutions to reduce maternal and neonatal mortality and build research capacity in Sierra Leone (NIHR133232) [Internet]. [Cited 2022 February 22]. Available from: https://fundingawards.nihr.ac.uk/award/NIHR133232

### O47 Implementation strategies to increase smoking cessation treatment provision in primary care: a systematic review of observational studies

#### Bernadett E Tildy^1,2^, Ann McNeill^1,2^, Parvati R Perman-Howe^1,2^, Leonie S Brose^1,2^

##### ^1^Addictions Department, King’s College London, London, United Kingdom; ^2^Shaping Public hEalth poliCies To Reduce ineqUalities and harm (SPECTRUM) Consortium, United Kingdom

###### **Correspondence:** Bernadett E Tildy (bernadett.tildy@kcl.ac.uk)

*Implementation Science 2023*, **18(Suppl 1):**O47

**Background**

Controlled trials have found some evidence for the efficacy of interventions aiming to increase the provision of smoking cessation treatment in primary care settings [1], but we need ‘real-world’ evidence, where implementation strategies [2] are implemented without researcher input. Aim: To identify ‘real-world’ implementation, effectiveness and cost-effectiveness of implementation strategies aiming to increase smoking cessation treatment provision in primary care, and any perceived facilitators and barriers for effectiveness.

**Method**

Seven databases, and three grey literature sources were searched from inception to April 2021. Studies were included if they evaluated implementation on a national or state-wide scale, contained practitioner performance and patient smoking outcome measures. Studies were assessed using the Risk Of Bias In Non-randomized Studies of Interventions (ROBINS-I) tool. A narrative synthesis was conducted using the ERIC compilation [3,4] and CFIR [5].

**Results**

Of 49 included papers, half were of moderate/low risk of bias. The implementation strategies identified involved utilising financial strategies, changing infrastructure, training and educating stakeholders, and engaging consumers. The first three strategies increased the provision of cessation advice in primary care but no intervention had high-quality evidence of impact on patient smoking cessation. No studies assessed cost-effectiveness. External policies/incentives (wider tobacco control measures and funding for public health and cessation clinics) were key facilitators. Time and financial constraints, lack of free cessation medications and follow-up, deprioritisation and unclear targets in primary care, lack of knowledge of healthcare professionals, and unclear messaging to patients about cessation were key barriers.

**Conclusion**

Some implementation strategies increased the rate of delivery of cessation advice in primary care, but there was no high-quality evidence showing an increase in quit attempts or smoking cessation. Barriers to effectiveness identified in this review should be reduced. More pragmatic approaches are recommended, such as ‘hybrid effectiveness-implementation designs’, and ‘Multiphase Optimization Strategy’ (MOST) [6].

**Systematic Review Registration:** PROSPERO: CRD42021246683

**Trial Registration:** Non applicable

**Consent to publish**

Non applicable

**References**

1. Lindson N, Pritchard G, Hong B, Fanshawe TR, Pipe A, Papadakis S. Strategies to improve smoking cessation rates in primary care. Cochrane Database of Systematic Reviews. 2021 Sep 6;2021(9).
2. Proctor EK, Powell BJ, McMillen JC. Implementation strategies: recommendations for specifying and reporting. Implementation Science. 2013;8(1):139.
3. Waltz TJ, Powell BJ, Matthieu MM, Damschroder LJ, Chinman MJ, Smith JL, et al. Use of concept mapping to characterize relationships among implementation strategies and assess their feasibility and importance: results from the Expert Recommendations for Implementing Change (ERIC) study. Implementation Science. 2015;10(1):109.
4. Powell BJ, Waltz TJ, Chinman MJ, Damschroder LJ, Smith JL, Matthieu MM, et al. A refined compilation of implementation strategies: results from the Expert Recommendations for Implementing Change (ERIC) project. Implementation Science. 2015;10(1):21.
5. Damschroder LJ, Aron DC, Keith RE, Kirsh SR, Alexander JA, Lowery JC. Fostering implementation of health services research findings into practice: a consolidated framework for advancing implementation science. Implement Sci. 2009 Aug;4:50.
6. Collins LM, Baker TB, Mermelstein RJ, Piper ME, Jorenby DE, Smith SS, et al. The multiphase optimization strategy for engineering effective tobacco use interventions. Ann Behav Med. 2011 Apr;41(2):208–26.

### P48 Result Of a feasibility hybrid II randomised controlled trial of volunteer ‘Health Champions’ supporting people with serious mental illness manage their physical health

#### Julie Williams^1^, Ray McGrath^2,5^, Karen Ang^3,5^, Isobel Mdudu^2^, Fiona Gaughran^2^, Ubong Akpan^2^, Errol Green^2^, Ioannis Bakolis^3^, Jorge Arias de la Torre^3^, Andy Healey^4^, Mariana Pinto da Costa^2^, Natalia Stepan^5^, Zarnie Khadjesari^6^, Euan Sadler^7^, Nick Sevdalis^1^ on behalf of the IMPHS study group

##### ^1^Centre for Implementation Science, King's College London, London, SE5 8AF, UK; ^2^South London and Maudsley NHS Foundation Trust, London, SE5 8AZ; ^3^Department of Biostatistics and Health Informatics, King's College London, SE5 8AF, UK; ^4^King's Health Economics, King's College London, SE5 8AF, UK; ^5^King's Health Partners Mind and Body Programme, London, SE1 9NT, UK; ^6^Behavioural and Implementation Science (BIS) Research Group, University of East Anglia, UK; ^7^Department of Nursing, Midwifery and Health, School of Health Sciences, University of Southampton, UK

###### **Correspondence:** Julie Williams (julie.williams@kcl.ac.uk)

*Implementation Science 2023*, **18(Suppl 1):**P48

**Background**

People with severe mental illness (SMI) such as schizophrenia are more likely to have physical health comorbidities than the general population. Interventions are needed to address this. Volunteers can bring a different and valued experience to supporting people with SMI. We report on a feasibility hybrid trial of an intervention called ‘Health Champions’ in which volunteers are trained to support individuals with their physical health.

**Method**

The study is a feasibility randomised Hybrid II trial. Health Champions provided weekly one to one support for up to nine months.

Our primary effectiveness outcome is physical health related quality of life and we also collected data on other related clinical and social outcomes. We collected data on clinical effectiveness at baseline and at the end of the intervention. We are conducting interviews with Health Champions and participants at the end of the intervention to understand their experience of the intervention and to evaluate the implementation challenges and collecting standardised Implementation Science measures. We are collecting data on the costs of the intervention as part of the economic evaluation.

**Results**

The intervention started during COVID and has been delivered both online and face-to face depending on the precautions at the time. To-date, we have recruited 48 participants, with 27 in the intervention arm and 21 in the control arm. We are still collecting data and will give an update on the results so far.

**Conclusion**

We will use the data collected to understand whether the Health Champions intervention is implementable, what the implementation challenges are, whether clinical and implementation outcomes can be collected and indicate any differences between trial arms, and whether the intervention is cost effective. We will use these results to decide on whether to undertake a larger trial and/or to recommend the intervention as part of routine care.

**Trial Registration:** Non applicable

**Consent to publish**

Non applicable

### O49 Evidence gap map on contextual analysis in implementation science

#### Juliane Mielke^1^, Thekla Brunkert^1,2^, Franziska Zúñiga^1^, Michael Simon^1^, Leah L. Zullig^3,4^, Sabina De Geest^1,5^

##### ^1^Institute of Nursing Science, Department Public Health, University of Basel, Basel, Switzerland; ^2^University Department of Geriatric Medicine FELIX PLATTER, Basel, Switzerland ^3^Center for Innovation to Accelerate Discovery and Practice Transformation (ADAPT) Durham, NC, USA; ^4^Department of Population Health Sciences, school of Medicine, Duke University, Durham, NC, USA; ^5^Academic Center for Nursing and Midwifery, Department of Public Health and Primary Care, KU Leuven, Leuven, Belgium

###### **Correspondence:** Juliane Mielke (juliane.mielke@unibas.ch)

*Implementation Science 2023*, **18(Suppl 1):**O49

**Background**

Understanding context is essential for successful and sustainable intervention implementation [1]. However, a lack of standardised methodological approaches for contextual analysis limits the assessment and leads to inconsistent reporting of context [2]. We systematically reviewed intervention implementation studies to map and evaluate current methodological approaches to contextual analysis.

**Method**

Applying a stepwise evidence gap map (EGM) approach, we empirically developed a search strategy to identify intervention implementation studies in PubMed (2015-2020) [3,4]. From a random sample (20%) of articles per year we assessed those in detail that reported on contextual analysis. Data extraction, analysis and evaluation was guided by the Basel Approach for CONtextual ANAlysis (a six-step guidance for contextual analysis) and the Context and Implementation of Complex Interventions (CICI) framework [1]. We created colour coded tables and visual maps to provide an overview on all relevant findings.

**Results**

We identified 15,286 intervention implementation studies and protocols, of which 3017 were screened for inclusion. Finally, 110 studies were included, with 24 (22%) reporting on contextual analysis.

Only one study used a framework explicitly guiding contextual analysis. Twenty-two studies focused on the meso-level (i.e., organisational characteristics) with socio-cultural aspects most frequently being studied. Commonly applied methods included surveys (n=15) and individual interviews (n=13), with ten studies reporting a mixed-methods analysis. In 18 studies, contextual information was used to inform subsequent project phases (e.g., intervention development/adaption, selecting implementation strategies); nine studies assessed influences of context on implementation and effectiveness outcomes.

**Conclusion**

This study provides an overview on current methodological approaches to contextual analysis while highlighting their gaps. The huge heterogeneity identified turns contextual analyses into “black boxes”. We strongly recommend taking concerted actions to further develop and test robust methodologies for contextual analysis and consistent reporting (e.g., following BANANA), to increase the quality and consistency of implementation science research.

**Trial Registration:** Non applicable

**Consent to publish**

Non applicable

**References**

1. Pfadenhauer LM, Gerhardus A, Mozygemba K, Lysdahl KB, Booth A, Hofmann B, Wahlster P, Polus S, Burns J, Brereton L et al: Making sense of complexity in context and implementation: the Context and Implementation of Complex Interventions (CICI) framework. Implement Sci 2017, 12(1):21.
2. Rogers L, De Brún A, McAuliffe E: Defining and assessing context in healthcare implementation studies: a systematic review. BMC Health Serv Res 2020, 20(1):591.
3. Hausner E, Waffenschmidt S, Kaiser T, Simon M: Routine development of objectively derived search strategies. Syst Rev 2012, 1(1):19.
4. Snilstveit B, Bhatia R, Rankin K, Leach B: 3ie evidence gap maps: a starting point for strategic evidence production and use, 3ie Working Paper 28. In. New Delhi: International Initiative for Impact Evaluation (3ie); 2017.

### O50

#### Withdrawn

### P51 Limiting and facilitating contextual factors impacting efforts to address gender norms underpinning female child marriage: a comparative case study of the implementation of the national strategy to end child marriage in Nigeria (2016-2021) and the national plan of action to end child marriage (2018-2030) in Bangladesh

#### Kelechi Udoh

##### Department for Health, 1 West, University of Bath, Claverton Down, Bath, United Kingdom

###### **Correspondence:** Kelechi Udoh (khu20@bath.ac.uk)

*Implementation Science 2023*, **18(Suppl 1):**P51

**Background**

Elimination of Female Child Marriage (FCM) remains a global health priority because FCM is associated with adverse health outcomes like teenage pregnancy and corresponding higher risks of puerperal endometritis, eclampsia, and systemic infections [1]. In 2016, the National Strategy to End Child Marriage in Nigeria (NSECMN) was introduced while in 2018, the National Plan of Action to End Child Marriage (NPAECM) was launched in Bangladesh, to amongst other priorities, address gender norms which evidence suggests remains the most potent and important factor underpinning FCM in both countries [2-4]. Despite these efforts, by 2021, the FCM rates in Nigeria and Bangladesh remained persistently high at 44% and 66%, respectively, which provided impetus for an analysis which would inform future endeavors to address the problem, in both countries [5,6].

**Method**

Guided by the Consolidated Framework for Implementation Research (CFIR), this study analyzed the contextual factors impacting NSECMN and NPAECM’s efforts at addressing gender norms underpinning FCM, using a document analysis method [7].

**Results**

Nigeria and Bangladesh’s spending on social programs (including for the implementation of both policies) were low during the reference period [8,9]. As such, financial and human resource support from international donors and non-governmental organizations ensured implementation feasibility [10,11]. While in both countries, the policies were backed by legislation, weak implementation remained a challenge [12,13]. This challenge was exacerbated by Nigeria and Bangladesh's multilateral legal systems which prevented the government from restricting FCM conducted under Islamic or customary laws [12,13]. Furthermore, in both countries, poverty fostered the norm of dowry payment, which promoted FCM [14,15].

**Conclusion**

Both countries need to increase national spending on FCM policy implementation, to reduce overreliance on international actors. They also need to introduce legislation that mandates adherence to civil law and adopt a holistic approach that ensures FCM policies are implemented in coordination with poverty alleviation programs.

**Trial Registration:** Non applicable

**Consent to publish**

Non applicable

**References**

1. WHO. Adolescent pregnancy [Internet]. Who.int. World Health Organization: WHO; 2020. Available from: https://www.who.int/news-room/fact-sheets/detail/adolescent-pregnancy
2. National strategy to end child marriage in Nigeria (2016-2021) [Internet]. Girls Not Brides. [cited 2022 Mar 30]. Available from: https://www.girlsnotbrides.org/learning-resources/resource-centre/national-strategy-end-child-marriage-nigeria-2016-2021/ [cited 2022 Mar 30].
3. National Action Plan to End Child Marriage (2018-2030). Ministry of Women and Children Affairs (MWCA)
4. Kohno A, Techasrivichien T, Suguimoto SP, Dahlui M, Nik Farid ND, Nakayama T. Investigation of the key factors that influence the girls to enter into child marriage: A meta-synthesis of qualitative evidence. Jong J, editor. PLOS ONE. 2020 Jul 17;15(7):e0235959.
5. Musa SS, Odey GO, Musa MK, Alhaj SM, Sunday BA, Muhammad SM, et al. Early marriage and teenage pregnancy: The unspoken consequences of COVID-19 pandemic in Nigeria. Public Health in Practice. 2021 Nov;2:100152.
6. Highest child marriage prevalence worldwide by country [Internet]. Statista. Available from: https://www.statista.com/statistics/1226532/countries-with-the-highest-child-marriage-rate/
7. Damschroder LJ, Aron DC, Keith RE, Kirsh SR, Alexander JA, Lowery JC. Fostering implementation of health services research findings into practice: a consolidated framework for advancing implementation science. Implementation Science. 2009 Aug 7;4(1).
8. Social Protection Sector Review in Nigeria [Internet]. www.ilo.org. 2019 [cited 2022 Mar 30]. Available from: https://www.ilo.org/africa/about-us/offices/abuja/WCMS_718388/lang%2D%2Den/index.htm
9. Social Safety Nets in Bangladesh Help Reduce Poverty and Improve Human Capital [Internet]. World Bank. Available from: https://www.worldbank.org/en/news/feature/2019/04/29/social-safety-nets-in-bangladesh-help-reduce-poverty-and-improve-human-capital
10. Improving Choices: Adolescent Sexual and Reproductive Health in Nigeria | Nigeria [Internet]. Save the Children | Nigeria. 2021 [cited 2022 Mar 30]. Available from: https://nigeria.savethechildren.net/news/improving-choices-adolescent-sexual-and-reproductive-health-nigeria
11. Because I am a Girl [Internet]. Plan International. [cited 2022 Mar 30]. Available from: https://plan-international.org/how-we-work/because-i-am-a-girl/
12. Akter S, Williams C, Talukder A, Islam MN, Escallon JV, Sultana T, et al. Harmful practices prevail despite legal knowledge: a mixed-method study on the paradox of child marriage in Bangladesh. Sexual and Reproductive Health Matters. 2021 Feb 24;29(2):1885790.
13. Obaje HI, Okengwu CG, Uwimana A, Sebineza HK, Okorie CE. Ending Child Marriage in Nigeria: The Maternal and Child Health Country-Wide Policy. Journal of Science Policy & Governance. 2020 Sep 30;17(01).
14. Mobolaji JW, Fatusi AO, Adedini SA. Ethnicity, religious affiliation and girl-child marriage: a cross-sectional study of nationally representative sample of female adolescents in Nigeria. BMC Public Health. 2020 Apr 29;20(1).
15. Bhowmik J, Biswas RK, Hossain S. Child Marriage and Adolescent Motherhood: A Nationwide Vulnerability for Women in Bangladesh. International Journal of Environmental Research and Public Health. 2021 Apr 12;18(8):4030.

### P52

#### Withdrawn

### O53 The Stanford Lightning Report: A pragmatic methodological approach for rapid qualitative synthesis

#### Cati Brown-Johnson, Nadia Safaeinili, Dani Zionts, Laura M. Holdsworth, Jonathan G. Shaw, Steven M. Asch, Megan Mahoney, Marcy Winget

##### Division of Primary Care and Population Health, Stanford University School of Medicine, Palo Alto, CA, 94619, USA

###### **Correspondence:** Cati Brown-Johnson (catibj@stanford.edu)

*Implementation Science 2023*, **18(Suppl 1):**O53

**Background**

A rapidly evolving healthcare implementation requires methods and tools to facilitate prompt communication with stakeholders while maintaining methodological rigor. The Stanford Lightning Report addresses these gaps with a methodological approach and flexible framework that innovates on debriefing techniques from manufacturing, enabling rapid feedback to healthcare partners.

**Method**

The Lightning Report method includes:

1. Pre-planning with evaluation partners to integrate emerging areas of interest into pre- existing collection tools.
2. Rapid synthesis. Structured research notes surface themes and unexpected findings. Researchers discuss notes/memos, and synthesize findings using Plus/Delta debriefing, adapted from Lean pedagogies.
3. Lightning Report creation. Components include executive summary, status of data collection, and findings that reflect Plus/Delta: what is going well with implementation, improvement opportunities and what needs to change, and suggested actions (“Insights”).

We assessed stakeholder perceptions of the value of the Lightning Report with a confidential feedback survey.

**Results**

We have used the Lightning Report in 20+ studies and quality improvement projects, in academic medicine, government health, and community. Stakeholders they are valuable, easy to understand, shared with colleagues, addressing important issues, and often influencing initiative implementation. Suggestions include wanting “larger number of completed interviews” and validation against systematic coding of transcripts. One healthcare partner reported that before Lightning Reports, they “got so little information during the first 3 to 4 years that we were unable to take corrective action that would help....”

**Conclusion**

The Stanford Lightning Report approach bridges the chasm between data collection and full data analysis/results publication. It can be rapidly developed from data to deliverable, is highly valued by partners, and generates stakeholder trust.

**Trial Registration:** Non applicable

**Consent to publish**

Non applicable

### P54

#### Withdrawn

### P55 What interventions should we implement in England’s mental-health services? The Mental-Health Implementation Network (MHIN) mixed-methods approach to rapid prioritisation

#### Shalini Ahuja, Lawrence Phillips, Christine McDonald, Caroline Smartt, Andrée le May, John Gabbay, Tina Coldham, Sarah Rae, Laura Fischer, Nick Sevdalis, Annette Boaz, Sarah Robinson, Fiona Gaughran, Zoe Lelliott, Peter Jones, Graham Thornicroft, Jayati-Das Munshi, Colin Drummond, Jesus Perez, Peter Littlejohns

##### NIHR ARC National Mental Health Implementation Network, Centre for Implementation Science, Health Service and Population Research Department, Institute of Psychiatry, Psychology and Neuroscience, Kings College London

###### **Correspondence:** Shalini Ahuja (shalini.ahuja@kcl.ac.uk)

*Implementation Science 2023*, **18(Suppl 1):**P55

**Background**

Setting mental health priorities helps researchers, policy makers, and service funders improve mental health services. In the context of a national mental health implementation programme in England, this study aims to provide a list of mental-health priority topics ripe for implementation, as well as a collection of adaptable methods and tools to help determine such priorities in future.

**Method**

A mixed-methods research design was used for a three-step prioritisation approach involving desk reviews, expert consultations and data triangulation. Groups with diverse expertise, including experts by experience, worked together to increase decision-making quality by engaging in deliberative discourse and modelling. A multi-criteria decision analysis (MCDA) model was used to combine participants' varied opinions, data and judgments about the data's relevance to the issues at hand during a decision conferencing workshop where the priorities were finalised.

**Results**

The study identified six mental-health priority topic areas for services: mental-health inequities, child and adolescent mental health, integration of mental and physical health, caregiver support and multi-morbidities, including mental health and drug misuse.

**Conclusion**

We report an inclusive attempt to ensure that the list of mental-health service priorities agrees with perceived needs on the ground and focuses on evidence-based interventions. Other fields of healthcare may also benefit from this methodological approach if they need to make rapid health-prioritisation decisions*.*

**Trial Registration:** Non applicable

**Consent to publish**

Non applicable

### P56 NIHR GHRG: CRIBS (Capacity. Research. Innovation. Building maternity Systems). Implementation of simple, scalable innovations & research capacity building to improve maternal health in Sierra Leone

#### Cristina Fernandez Turienzo^1^, Alexandra Ridout^1^ , Mangenda Kamara^2^, Lucy November^2^, Prince T Williams^3^, Frances Moses^4^, Venetia Goodhart^2^, Suzanne Thomas^2^, Simren Herm-Singh^2^; Katy Kuhrt^1^, Betty Sam^4^; Paul T Seed^1^, Kate Brahman^1^, Jane Sandall^1^, Sahr Gevao^5^, Andrew H Shennan^1^, on behalf of NIHR CRIBS Group

##### ^1^Department of Women and Children’s Health, Faculty of Life Sciences & Medicine, King’s College London, UK; ^2^Welbodi Partnership, Sierra Leone; ^3^Lifeline Nemeniah Projects, Sierra Leone; ^4^Ministry of Health and Sanitation, Sierra Leone; ^5^University of Sierra Leone, Sierra Leone

###### **Correspondence:** Cristina Fernandez Turienzo (cristina.fernandez_turienzo@kcl.ac.uk)

*Implementation Science 2023*, **18(Suppl 1):**P56

**Background**

This National Institute for Health Research (NIHR) Global Health Research Group builds on multidisciplinary research partnerships spanning the last five years, formalising the collaboration between King’s College London and the University of Sierra Leone, and brings collaborations with many partners, including the Ministry of Health and Sanitation (MoHS) in SL, iNGO Welbodi Partnership, Lifeline Nehemiah Project, and the National Midwifery Schools. The overall aim is to develop and implement simple, scalable innovations to reduce maternal and perinatal mortality and build research capacity and expertise in Sierra Leone.

**Method**

We developed a programme of work addressing locally identified maternal health challenges using local pilot data to inform assumptions and feasibility. We created several projects with the aim of improving health outcomes through implementation, practice and policy, alongside sustainably strengthening research capacity and capability. MRC framework, RE-AIM and Proctor’s outcomes will guide nested implementation evaluations.

**Results**

The main workstreams include:

- A stepped-wedge, hybrid implementation-effectiveness randomised controlled trial to evaluate the implementation and real-world scale up of the CRADLE device and training across rural Sierra Leone, with the aim of providing a blueprint for scale-up worldwide.
- A randomised cluster pilot trial to assess the feasibility and implementation of a locally designed and community based intervention providing mentoring from pregnancy through to one-year post-birth for adolescent girls (addressing the social factors of stigma, abandonment and poor maternal health outcomes)
- An evaluation of shock index as a predictor of adverse outcomes secondary to haemorrhage and sepsis in pregnant women (compared to conventional vital signs monitoring)
- A validation of a point-of-care creatinine device to detect acute kidney injury in pregnancy, a preventable cause of maternal morbidity and mortality.
- Build research capacity and expertise by supporting local PHD students, MPH students and early career researchers.

**Conclusion**

The University of Sierra Leone and King’s College London, with support from in country collaborators, have partnered to build maternal health implementation and evaluation research and expertise where it is needed most. Close partnership and planning will promote uptake and success, strengthen institutional capacity and create a platform for advancement in health projects and services, across all cadres of maternal health provider.

**Trial Registration:** Non applicable

**Consent to publish**

Non applicable

### O57 CRADLE 5: Evaluating the national scale-up of the CRADLE Vital Sign Alert device in Sierra Leone. Helping pregnant women get to the right place at the right time

#### Alexandra Ridout^1^, Simren Herm Singh^2^, Francis Moses^3^ , Kellie Koroma^2^, Venetia Goodhart^2^, Sister Betty Sam^2^, Matron Mariama Momoh^3^, Katy Kuhrt^1^, Francis Smart^3^, Sartie Kenneh^3^, Jane Sandall^1^, Andrew Shennan^1^ on behalf of NIHR CRIBS Global Health Group

##### ^1^King’s College London, UK; ^2^Welbodi partnership, Freetown, Sierra Leone; ^3^Ministry of Health and Sanitation, Sierra Leone

###### **Correspondence:** Alexandra Ridout (alexandra.ridout@kcl.ac.uk)

*Implementation Science 2023*, **18(Suppl 1):**O57

**Background**

The CRADLE Vital Sign Alert is an easy-to-use, accurate device that measures blood pressure and pulse, with an incorporated traffic-light early warning system and training package. CRADLE was associated with reduced rates of maternal death (RR 0.37 [95% CI 0.25 to 0.55], p<0.0001) and eclampsia (RR 0.56 [95% CI 0.41 to 0.67], P<0.0001) when introduced into an urban centre in Sierra Leone. This evidence produced political buy-in for scale-up, which was piloted in half of the country. The WHO Expand Net framework was used to design the key elements. Effectiveness of implementation strategies, fidelity and feasibility were positively evaluated.

Funding has now been obtained as part of an NIHR Global Health Research Group (CRIBS) to determine the impact, adoption and sustainability of CRADLE scale-up into routine maternity care in Sierra Leone.

**Method**

A randomised effectiveness-implementation type 2 trial will evaluate the intervention across eight rural districts in a stepped-wedged design. All women identified as pregnant or within 6 weeks postpartum, presenting for maternity care at any level of government facility, will be eligible to participate. Primary outcome data (composite of maternal death, eclampsia and hysterectomy per 10,000 deliveries and stillbirth per 1,000 deliveries) will be collected. Implementation of the intervention will be evaluated via a mixed-methods approach. Process evaluation measures will be analysed using the RE-AIM framework. Measures and tools have been co-designed and optimised during pilot work. An offline mobile phone application has been designed to capture reach using GPS. A nested evaluation of experiences of care and mechanisms, including impact of referral patterns and clinical care escalation is planned. Sustainability, including a policy lab, will be conducted.

**Discussion**

This trial will demonstrate the potential impact of CRADLE on reducing neonatal and maternal mortality and morbidity in low-resource settings. It is anticipated that its relatively low cost and ease of integration into existing health systems will be of significant interest to local, national and international health policy-makers.

**Trial Registration:** ISRCTN94429427. Registered April 2022.

**Consent to publish**

Non applicable

